# The impact of mistranslation on phenotypic variability and fitness

**DOI:** 10.1111/evo.14179

**Published:** 2021-02-02

**Authors:** Laasya Samhita, Parth K Raval, Godwin Stephenson, Shashi Thutupalli, Deepa Agashe

**Affiliations:** ^1^ National Centre for Biological Sciences Tata Institute of Fundamental Research Bangalore India; ^2^ International Centre for Theoretical Sciences Tata Institute of Fundamental Research Bangalore India

**Keywords:** Fitness, heterogeneity, mistranslation, phenotypic variation, variability

## Abstract

Phenotypic variation is widespread in natural populations, and can significantly alter population ecology and evolution. Phenotypic variation often reflects underlying genetic variation, but also manifests via non‐heritable mechanisms. For instance, translation errors result in about 10% of cellular proteins carrying altered sequences. Thus, proteome diversification arising from translation errors can potentially generate phenotypic variability, in turn increasing variability in the fate of cells or of populations. However, the link between protein diversity and phenotypic variability remains unverified. We manipulated mistranslation levels in *Escherichia coli*, and measured phenotypic variability between single cells (individual‐level variation), as well as replicate populations (population‐level variation). Monitoring growth and survival, we find that mistranslation indeed increases variation across *E. coli* cells, but does not consistently increase variability in growth parameters across replicate populations. Interestingly, although any deviation from the wild‐type (WT) level of mistranslation reduces fitness in an optimal environment, the increased variation is associated with a survival benefit under stress. Hence, we suggest that mistranslation‐induced phenotypic variation can impact growth and survival and has the potential to alter evolutionary trajectories.

Non‐genetic phenotypic variability has long fascinated biologists, not least due to its potential evolutionary consequences. Several aspects of such variation have been analyzed from distinct perspectives. Perhaps the best‐studied form of non‐genetic variation is phenotypic plasticity, when individual phenotype changes in response to the local environment. Such plasticity is sometimes adaptive in animals and plants, and has clear consequences for population as well as community ecology and evolution (reviewed in Bolnick et al. 2011; Raffard et al. 2019). In other cases, only some individuals in a population may respond to environmental change at a given point of time. The resulting heterogeneity in the population potentially represents an evolved bet‐hedging strategy, whereby different fractions of the population are better adapted to distinct environments (reviewed in Ackermann 2015). For instance, some cells in *Bacillus subtilis* populations form inactive spores in stressful conditions (Tan and Ramamurthi 2014), whereas others remain metabolically active. Under prolonged stress, the spores stand a better chance of survival; however, if the stress is transient, non‐spore formers divide more rapidly. Finally, rather than specific responses to environmental change, genetically identical cells may have distinct phenotypes due to “noise” arising from stochastic variation in gene expression or errors in transcription and translation (Drummond and Wilke 2009; Gout et al. 2013; Ackermann 2015; Carey et al. 2018). Such phenotypic heterogeneity has been well studied in microbial populations, although its evolutionary consequences are relatively poorly understood (Ackermann 2015; van Boxtel et al. 2017).

The evolutionary impacts of non‐genetic variation are usually reported either as divergent individual‐level outcomes (e.g., genetically identical cells with distinct phenotypes may have different reproductive success; Fig. 1a), or as altered mean population‐level parameters (e.g., heterogeneous populations may grow more slowly than homogeneous populations; Fig. 1b). Both are useful from an evolutionary perspective: growth and survival of individuals ultimately determine trait mean and variance within the population, and hence the outcome of selection. While it is clear that variability in single cell growth rates influences population growth, the impact and direction of this influence remains debated. For example, some models show that variability in single cell growth rates can decrease average population growth rate (Lin and Amir 2017), whereas other models find that increased noise in single cell growth rate results in higher mean population growth rate (Hashimoto et al. 2016). However, non‐genetic variation could alter not only average population performance, but also the variance in population‐level parameters. For instance, replicate populations with high individual‐level heterogeneity may have more variable average growth rates than replicate homogeneous populations (Fig. 1c). This may occur due to stochastic effects in each replicate; correlated mother and daughter cell division times across generations that mimic heritability and hence perpetuate across generations (Lin and Amir 2017); or due to divergent outcomes of interactions between individuals in each population. Thus, despite similar initial heterogeneity within each replicate, the population level outcomes may be divergent (right‐hand plot in Fig. 1c). Such effects on variance are important to measure, because the higher variance in evolutionary outcomes between populations reduces the repeatability (and hence predictability) of evolutionary dynamics. However, the potential impact of between‐individual phenotypic heterogeneity on the variance in population‐level parameters remains unexplored. Hence, it is unclear whether a mechanism that generates individual‐level variation (i.e. between cells or organisms) also consistently generates divergent population‐level outcomes.

**Figure 1:**
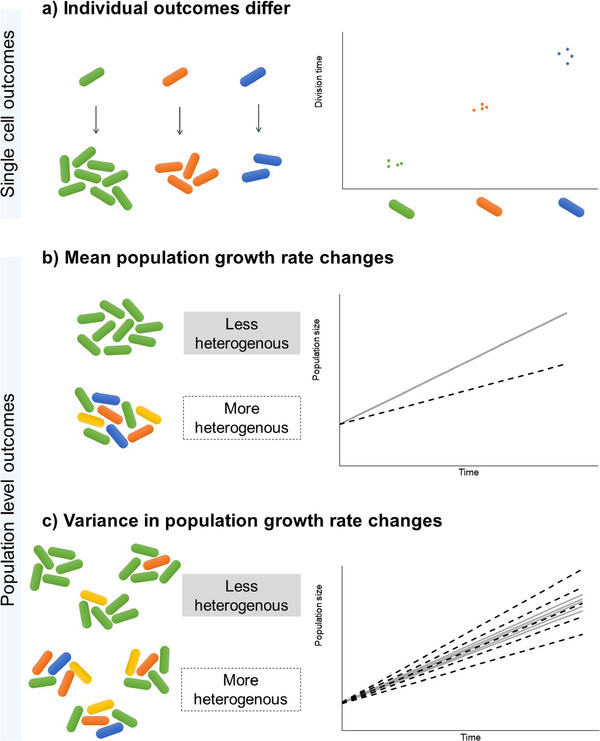
Possible impacts of individual or cell‐to‐cell heterogeneity on individuals and populations. (a) Cell to cell heterogeneity (indicated by cell color) causes variation in individual fitness parameters such as the rate of cell division. (b) Populations that have a large amount of cell to cell heterogeneity may grow more slowly, because of the phenotypic load generated by slower‐dividing cells. (c) Cell to cell heterogeneity can impact between‐population variance due to stochastic effects or diverse cell to cell interactions. As a result, replicate populations with high individual‐level heterogeneity may show more variable growth rates (spread around the mean value) than replicate homogeneous populations.

From a mechanistic perspective, translation errors are especially interesting because they are an inescapable aspect of the biology of all life forms, and they occur at a high rate. For instance, in *Escherichia coli*, about 10% of dihydrofolate reductase enzyme molecules differ from the native sequence of the protein (Ruan et al. 2008). The typical mistranslation rate is ∼1 in 10^4^ incorrect amino acids in a growing protein chain, increasing to as high as 1 in 10^3^ amino acids under stress (Ribas de Pouplana et al. 2014; Mordret et al. 2019). Such high error rates can generate significant proteome diversity (Nakahigashi et al. 2016; Mordret et al. 2019). Importantly, unlike many other forms of non‐genetic variability in microbes – such as spore formation and persister cells (Ackerman 2015) – mistranslation can generate continuous (rather than binary) phenotypic variation, allowing a more fine‐tuned response to diverse stresses. Such continuous variation in protein quality or quantity can reliably generate large phenotypic variability, as seen with the heat shock protein Hsp90 (Cowen and Lindquist 2005) and prions (Halfmann et al. 2012), which in turn can determine survival in a new environment (Novick and Weiner 1957) (reviewed in Samhita 2020). Therefore, it is speculated that mistranslation‐induced non‐genetic variation may generate substantial phenotypic variability, potentially altering the outcome of natural selection (Miranda et al. 2013; van Boxtel et al. 2017; reviewed in Samhita 2020).

However, postulating a general hypothesis about the evolutionary consequences of mistranslation‐induced variation requires consideration of multiple nuances. First, proteome diversity is visible to natural selection only if it leads to phenotypic diversity in traits that influence fitness. Given various buffering mechanisms driven by chaperones and the degradation of mistranslated products (Bratulic et al. 2015; Kalapis et al. 2015), protein diversity may not always generate phenotypic diversity. Hence, in the absence of this link, proteome diversity is of little evolutionary consequence. Second, the effects of mistranslation are inherently unpredictable and not heritable, weakening the potential for long‐term consequences. In microbes such as *E. coli*, proteome diversity has limited across‐generation persistence due to protein dilution at cell division. Hence, favorable mistranslated protein variants may never be sampled again, limiting their effect on evolutionary dynamics. Finally, in a constant optimal environment, populations should face stabilizing selection. This means that any mechanism that generates increased variability between individuals is likely to move them away from the optimal phenotype, creating a “phenotypic load.” Therefore, if mistranslation increases cell to cell variability, it is likely to be adaptive primarily under directional or disruptive selection, such as might be imposed in a new environment or under stress. In contrast, in a constant environment, mistranslation is more likely to be maladaptive. These limitations of the evolutionary consequences of mistranslation remain largely untested. Previous work shows that increased mistranslation can generate diversity in cell morphology and cell surface receptors (Bezerra et al. 2013; Miranda et al. 2013). However, experimental evidence directly linking mistranslation with phenotypic variation relevant to fitness is rare.

Here, we tested whether altering mistranslation levels in *E. coli* impacts variability at both single cell and population levels, in phenotypes relevant for growth and survival. We increased the basal level of mistranslation in wild type (WT) cells by introducing mutations in translation components or by changing the growth environment. Recently, we showed that generalized mistranslation increases mean population survival under specific stresses (Samhita et al. 2020). However, we had not explored whether mistranslation generates phenotypic variability that influences both cell and population fitness, and in optimal as well as stressful environments. Here, we find that mistranslation indeed increases phenotypic diversity in *E. coli* at the single cell level, and that suppressing mistranslation via hyper‐accurate ribosomes reduces this variability. However, increased single‐cell variability did not always affect variability in population level growth parameters. Importantly, while mistranslation‐associated variability is costly in optimal conditions, it increases survival under stress; and this effect is observed even with a transient increase in mistranslation. Thus, mistranslation indeed results in phenotypic diversification across cells, and this diversity is directly correlated with survival under stress.

## RESULTS

### MISTRANSLATION CONSISTENTLY INCREASES CELL‐TO‐CELL BUT NOT BETWEEN‐POPULATION VARIABILITY IN GROWTH AND DIVISION TIME

**Figure 2:**
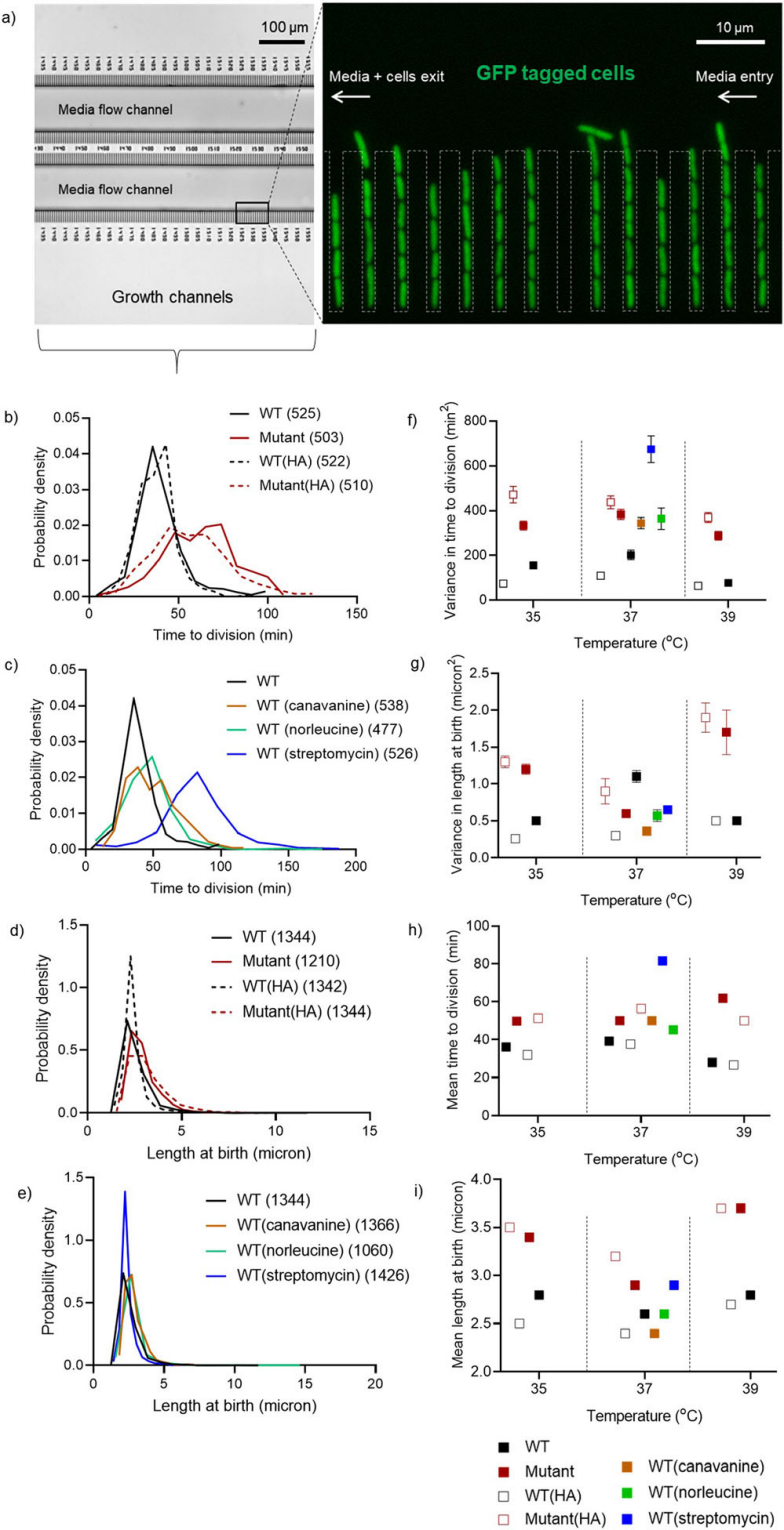
Mistranslation increases cell‐to‐cell variability in growth and division time: We injected ∼10^5^ cells of the indicated strains into a microfluidic device designed for single cell tracking, and monitored cell growth and cell length under the microscope. (a) Schematic of the microfluidics device showing GFP tagged *E. coli* single cells growing and dividing in channels within the device. (b–e) Probability density functions for cell length and division time of single cells as monitored in the microfluidics device. Wider distributions indicate greater cell‐to‐cell variation. Total number of cells (n) is indicated within parentheses in the key. WT = wild type; HA = hyper‐accurate. (f–i) Estimates of mean and variance of single cell division time and birth length obtained from bootstrap analysis (n = 10^6^), with SD values. In some cases, the SD value is too small to be visible.

To test the impact of mistranslation on phenotypic variation, we manipulated basal mistranslation levels and measured division time and cell length of GFP‐tagged, isolated single cells in a microfluidics device (Fig. 2a). Cell division time is a key proxy for fitness under optimal growth conditions, and changes in cell length are predictive of the physiological state of a cell (Wehrens et al. 2018). We genetically increased mistranslation levels in our WT *E. coli*, generating the “Mutant”: a strain with depleted initiator tRNA content that has increased mistranslation rate (Samhita et al. 2013). Conversely, we reduced mistranslation rate by introducing a mutation in the ribosomal protein S12 of both WT and Mutant strains, creating the strains WT(HA) and Mutant(HA) (“hyper‐accurate” strains, see Methods and Table 1). Other than these genetic manipulations, we also increased mistranslation rates by adding the amino acid analogs canavanine or norleucine, or the antibiotic streptomycin to the growth medium. In each case, we measured phenotypic variability across cells and across populations. Because data were not normally distributed, we compared distributions for spread around the median using the Fligner‐Killeen test. The comparison accounts for central tendency (median) as well as sample size, and reports whether two distributions have similar variability. To infer the direction of the difference, we used the range of the data in both distributions.

**Table 1:** Strains

Common name used in the text	Strain	Genotype/details	Reference
WT	KL16	*E. coli* K‐12, *thi1, relA1, spoT1*	(Low 1968)
Mutant	KL16*ΔZWV*	Derivative of KL16 lacking three initiator tRNA genes, *metZ*, *metW* and *metV*	(Samhita et al. 2012)
WT(HA)	KL16*rpsL*	Derivative of KL16 with an additional mutation in *rpsL* (K42R)	This study
Mutant(HA)	KL16*ΔZWVrpsL*	Derivative of KL16*ΔZWV* with an additional mutation in *rpsL* (K42R)	This study

As predicted, all methods of increasing mistranslation increased cell‐to‐cell variation in the time to division (Fig. 2b–c; Fligner Killeen test: Mutant >WT, *Χ* ^2^ = 9, *p* < 0.0001; WT_can_>WT, *Χ* ^2^ = 84.9, *p* < 0.0001; WT_nor_>WT, *Χ* ^2^ = 54.1, *p* < 0.0001; WT_strp_>WT, *Χ* ^2^ = 32.6, *p* < 0.0001; Table S1) and most led to increased cell size variability (Fig. 2d–e; Fligner Killeen test: Mutant>WT, *Χ* ^2^ = 86.9, *p* < 0.0001; WT_can_<WT, *Χ* ^2^ = 46.4, *p* < 0.0001; WT_nor_>WT, *Χ* ^2^ = 58.5, *p* < 0.0001; WT vs WT_strp_, ns, *Χ* ^2^ = 1.5, *p* = 0.2; Table S1). Conversely, reducing mistranslation via hyper‐accurate ribosomes reduced variability in cell size but not division time of the WT, and did not reduce either phenotype in the Mutant (Fig. 2b and d; see Table S1). Similar results were obtained at slightly different temperatures (35°C and 39°C; Fig. S2 and Table S1). In the analyses described above, for each strain, we pooled data across ∼60 channels of the microfluidics device (each with a single, original mother cell) and ∼600 divisions. To confirm that this pooling did not end up averaging differences across generations, we re‐analyzed data focusing only on the first three divisions of each mother cell (∼60 cells in total); and found similar results (Fig. S3). In addition, bootstrap analysis of each dataset at 37°C showed a high degree of convergence in the estimates of mean and variance in time to division and cell length (Fig. S4). Because each single cell effectively represents an independent cellular lineage in the mother machine, the bootstrapped samples represent multiple sets of “replicate populations.” All pairwise comparisons of means and variance from the bootstrap analysis were significant (Tukey's HSD accounting for multiple comparisons, adjusted *p*‐value < 0.001), indicating that the patterns reported above are robust (Fig. 2f–g). Thus, our microfluidics experiments show that mistranslation directly increases single‐cell phenotypic variability in growth and division time.

**Figure 3:**
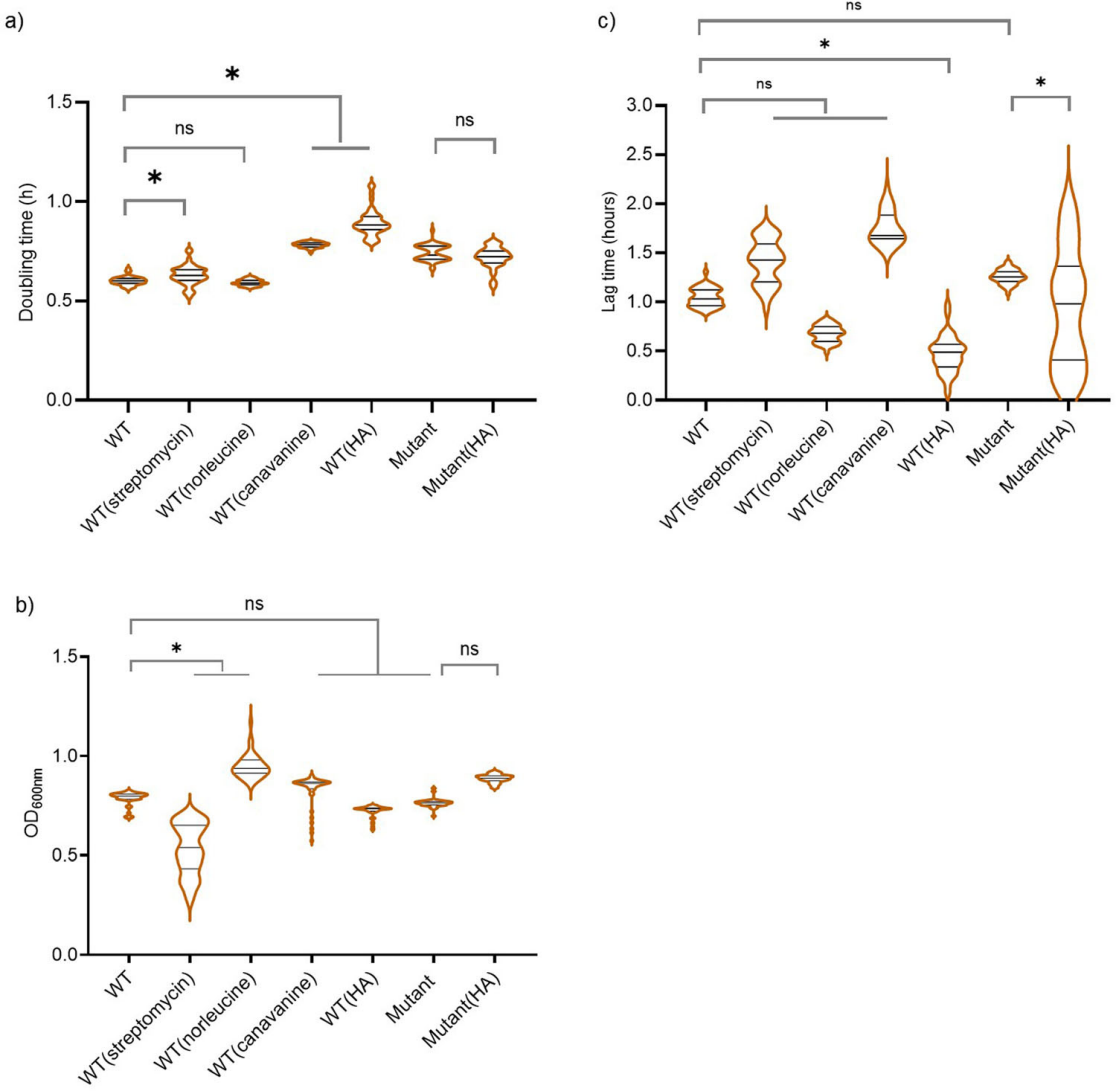
Mistranslation does not impact variability in growth parameters across replicate populations: Violin plots showing the distribution of three population growth parameters, estimated using ∼40 (37 to 44) biological replicates (populations) for each strain or growth condition. (a) Doubling time, (b) growth yield. and (c) lag time (time until culture reaches OD_600_ ∼0.02). Median, 25th and 75th quartiles are indicated by solid lines within each violin. The length of each violin corresponds to the range of the distribution. Asterisks indicate significant differences in variability. WT = wild type; HA = hyper‐accurate.

Next, we examined the consequences of mistranslation on variability at the population level. We compared growth curves of ∼40 replicate populations per strain/condition (Fig. S5), to test whether cell to cell variability manifests at the population level in growth parameters of mistranslating strains, as discussed in the Introduction. Our single cell measurements agree with previous observations of poor correlation between the division times of mother and daughter cells (Fig. S3) in *E. coli* (Hashimoto et al. 2016). For this reason, we hypothesized that mistranslation may not increase variability in population‐level growth parameters, unlike our observations across single cells. We measured four growth parameters: population aggregate doubling time, growth rate, growth yield, and lag time. Of these, population doubling time distributions can be compared directly with their respective single cell division time distributions, because they measure the same underlying trait. While we expect growth rate, growth yield, and lag time to be influenced by differences in division time and cell length, they do not correspond directly to the traits measured at the single‐cell level. In the case of doubling time, the results for population‐level growth indeed mirrored our observations for single cell variation: mistranslation level significantly affected between‐population variability in all cases except WT vs. WT(nor) and Mutant vs. Mutant (HA) (Fig. 3a). Interestingly, increasing mistranslation did not increase between‐population variability in growth rate, lag time, or growth yield except for the influence of streptomycin and norleucine on growth yield (Fig. 3, Fig. S6; Table S1). Contrary to expectation, reducing mistranslation through hyper‐accurate ribosomes also increased variability in the WT lag time and in all three parameters for the Mutant (Fig. 3; Table S1). Thus, in contrast to single‐cell variability, mistranslation did not consistently affect variability in population‐level growth parameters.

### MISTRANSLATION IS COSTLY UNDER NORMAL CONDITIONS

In addition to affecting variability in single‐cell growth parameters, altering mistranslation levels often incurred a cost. At the single cell level, median time to division increased significantly with higher mistranslation, although reducing mistranslation had no effect (e.g. WT: 36 min, Mutant: 62 min; Mann‐Whitney test, *U* = 45832, *p *< 0.001; Fig. 2b, c, h; Table S1). Increased mistranslation also increased cell length in most cases (Fig 2d, e, i; WT 2.4 μm versus Mutant 2.7 μm, Mann‐Whitney test, *U* = 586570, *p *< 0.0001; other comparisons in Table S1), whereas reducing WT mistranslation through hyper‐accurate ribosomes decreased cell length (WT 2.4 vs WT(HA) 2.2 μm, Mann‐Whitney test, *U* = 710144, *p *< 0.001). Increased cell length might partly account for greater division times of mistranslating strains, although we did not explicitly test this. Note that while increased cell length is associated with stressful conditions (Wehrens et al. 2018), it is not clear if longer cells are necessarily costly here, given the associated increase in biomass. These patterns at the single‐cell level were also reflected in population‐level parameters. Populations with either increased or decreased mistranslation relative to WT had longer doubling time, lower growth rate, and greater lag time for the most part; although growth yield did not change consistently (compare median values in Fig. 3a and c; also see Fig. S7, S8 and Table S1). Overall, mistranslating cells were longer and divided more slowly than the WT, and mistranslating populations showed slower growth; suggesting a cost of mistranslation.

### MISTRANSLATION INCREASES POPULATION SURVIVAL UNDER STRESS

**Figure 4:**
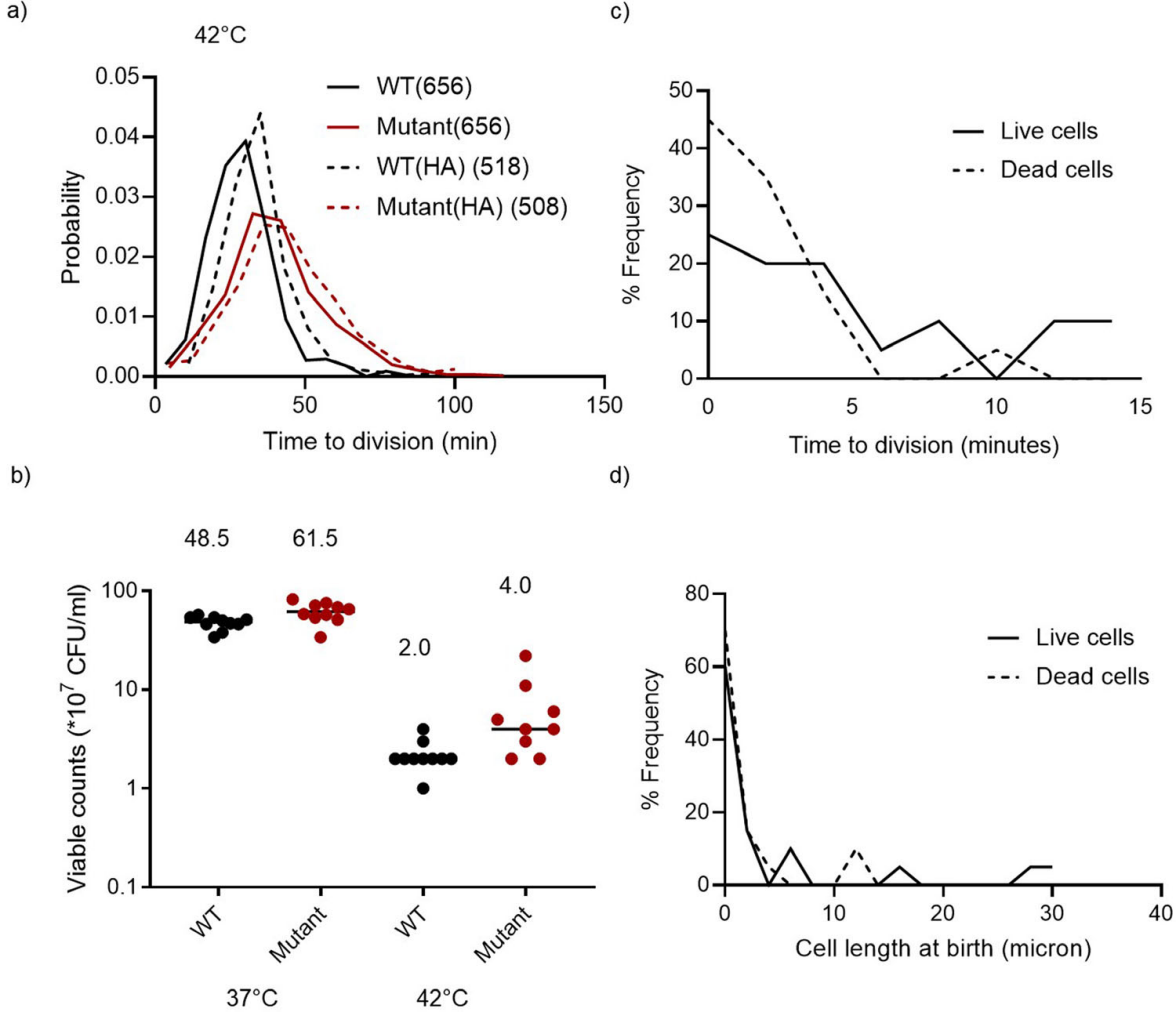
Mistranslation increases survival under stress: (a) Frequency distributions of division time of single cells of different strains monitored in the microfluidics device at 42°C. The center of each bin (class interval) is 5 units, and each such point is connected to the next one by a line. WT = wild type; HA = hyper‐accurate. (b) Total viable counts (estimated by dilution plating) in WT and Mutant cultures (n = 9) grown for 48 h (late stationary phase) at 37°C or 42°C. Numbers indicate median viable counts in each case. (c–d) Distribution of division time and cell length of single WT cells that subsequently either survived (live cells, n = 86) or died (dead cells, n = 31) after being starved of nutrients for ∼10 h, in saline. Overall, WT cells had a higher fraction of dead cells than the Mutant (see Results).

Although costly under normal conditions, previous studies suggest that mistranslation often confers a benefit under stress at the population level (reviewed in Mohler and Ibba 2017). We therefore examined the impact of two stresses – high temperature (42°C) and starvation (Koch 1971; van Elsas et al. 2011) – on single cell and on population growth parameters. Both WT and Mutant single cells divided faster at 42°C than at 37°C, but the increase was much larger in the Mutant (compare Fig. 4a vs. Fig. 2b; median division time WT (42°C): 28 min vs. WT (37°C): 36 min, Mann‐Whitney test, *U* = 89652, *p *< 0.0001; Mutant (42°C): 38 min versus Mutant (37°C): 62 min, *U* = 66956, *p *< 0.0001). Thus, the cost of mistranslation decreased at high temperature; but the Mutant still took longer to divide than the WT (median division time: Mutant 38 min vs. WT 28 min, Mann‐Whitney test, *U* = 123307, *p *< 0.0001). At 42°C, reducing mistranslation rate was also slightly costly for the Mutant (median division time: Mutant (HA) 40 min, Mutant 38 min, Mann‐Whitney test, *U* = 149832, *p* = 0.003), and more so for the WT (median division time WT(HA) 34 min >WT 28 min, Mann‐Whitney test, *U* = 118457, *p *< 0.0001; Fig. 4a). As at 37°C, parameter estimates from bootstrap analysis indicate that these results are robust (Fig. S9 and Table S1). Overall, an increase in temperature reduced the cost of slow growth and mistranslation (as measured by division time difference) in the Mutant, but did not give it a growth advantage over the WT. All else being equal, division time is a good measure of fitness in actively dividing cells; but once cells enter stationary phase, division rate decreases and growth rate no longer determines competitive fitness. We, therefore, examined longer‐term survival as a population level fitness measure, assessing total viable counts in 48 h (stationary phase) cultures exposed to high temperature. Across populations, mutant survivability was higher than WT at both 37°C and 42°C, with a stronger effect at 42°C (Fig. 4b; Table S1). Thus, mistranslation was either beneficial (or less costly compared to WT) for cells and populations exposed to high‐temperature stress.

Next, we tested cell survival under starvation stress. We introduced diluted overnight cultures in the microfluidics device, allowed cells to enter mid‐log phase and then added saline (0.85% NaCl) instead of growth medium, to test whether Mutant and WT had different rates of cell death in the absence of nutrients (where no cell division can occur). We did this across three independent experimental blocks, sampling ∼650 cells of WT and Mutant each in total (Fig. S10). After 10 h, in each block, a larger fraction of WT cells died (on average ∼19% WT cells died, while only ∼4% Mutant cells died, WT>Mutant, *t*‐test, *t* = 14.3, *p *< 0.0001); suggesting that the Mutant is more robust to a lack of nutrients. To test whether dead cells were more likely to have a specific cellular phenotype, we retrospectively measured the time to division and cell length of 31 dead cells and 86 live cells of WT from one block. Interestingly, both surviving and dead cells were drawn from across the original distribution of cell length and division time (Fig. 4c–d), indicating that survival was not linked to these specific aspects of cellular level heterogeneity. We could not do a similar analysis for the Mutant, due to the small number of dead cells. Our results suggest that mistranslation increases cell survival under starvation‐induced stress; but that the survival is not directly connected to the indicators of cellular phenotype that we measured.

**Figure 5:**
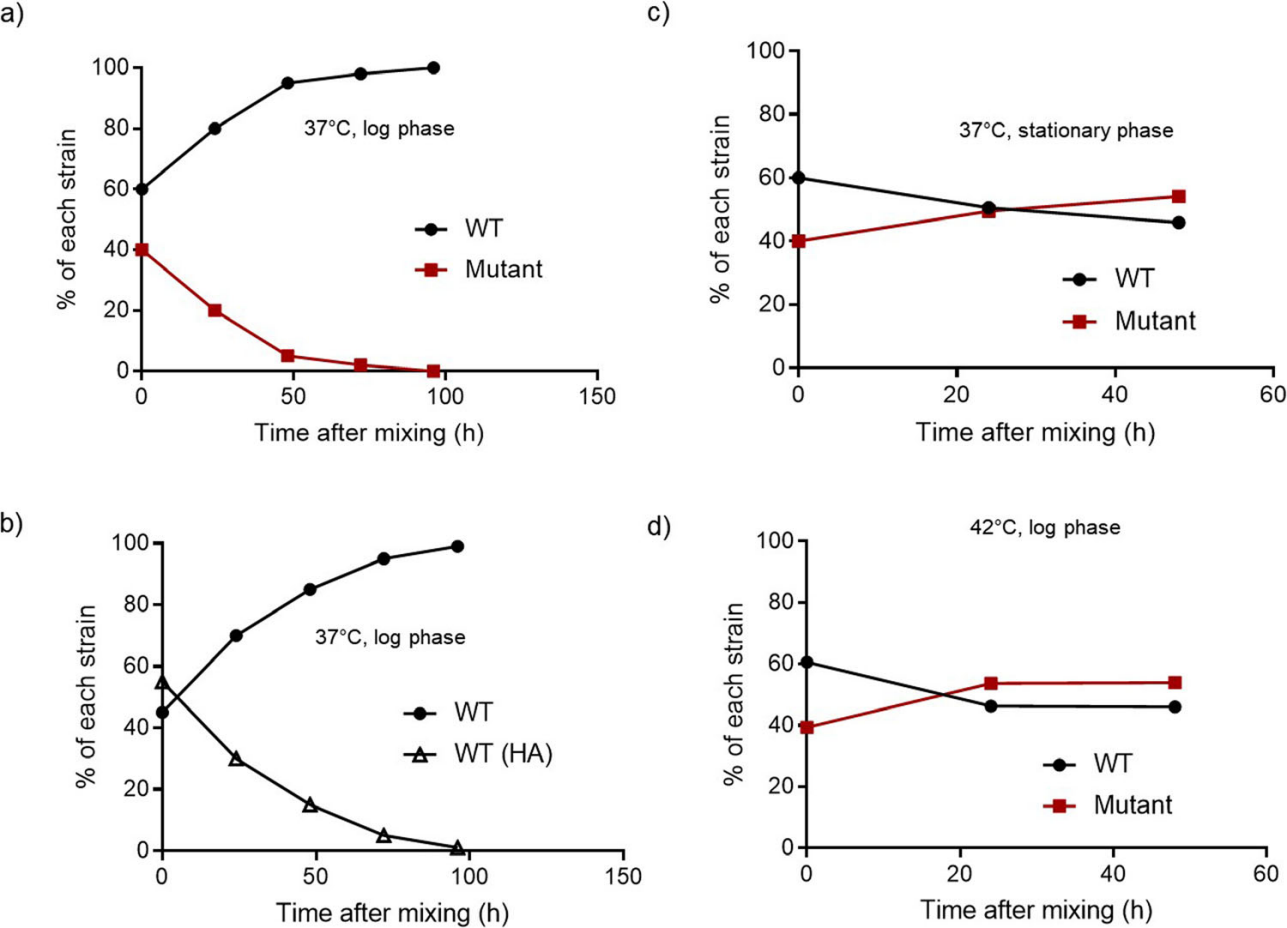
Mistranslation is costly under optimal conditions but beneficial under stress: (a–b) Cell survival as a function of time, during pairwise competition in the log phase of growth at 37°C. We mixed log‐phase cultures (OD_600_∼0.6) of two strains (as indicated) in LB, and plated aliquots on MacConkey's agar to estimate survival of each strain. Data for a representative block for each case are shown here; other blocks are shown in Fig. S10. (c) Cell survival as a function of time, during pairwise competition in the stationary phase of growth at 37°C. We allowed WT and Mutant cultures to grow independently for 48 h in LB medium and then mixed them to assess competition in late stationary phase. Data for a representative experimental block for each case are shown here; other blocks are shown in Fig. S10. (d) Cell survival as a function of time, during pairwise competition in the log phase of growth at 42°C.

Lastly, we assessed the impact of mistranslation on population survival using competitive fitness. We allowed WT to compete with its hyper‐accurate derivative or with the Mutant, using actively growing (log phase) or stationary phase cultures (under starvation) as a starting point. As expected from their relative log phase growth rates (Fig. 3a), WT outcompeted both the mutant and the hyper‐accurate strains (Fig. 5a and b; Fig. S11). However, when competing in stationary phase, Mutant had comparable or marginally higher fitness than the WT, both at 37°C and 42°C (Fig. 5c and d). Thus, both increasing or decreasing mistranslation levels in the WT imposed a fitness cost in nutrient‐rich conditions when rapid growth is favored. In contrast, under stress, cells with higher mistranslation rates could either co‐exist with or perform slightly better than cells with a lower mistranslation rate. Together, our results indicate that mistranslation is costly for growth under optimal conditions, but is often beneficial for survival under stress, at the single cell as well as population levels.

### THE DEGREE OF MISTRANSLATION DOES NOT CORRELATE WITH BETWEEN‐POPULATION VARIABILITY

**Figure 6:**
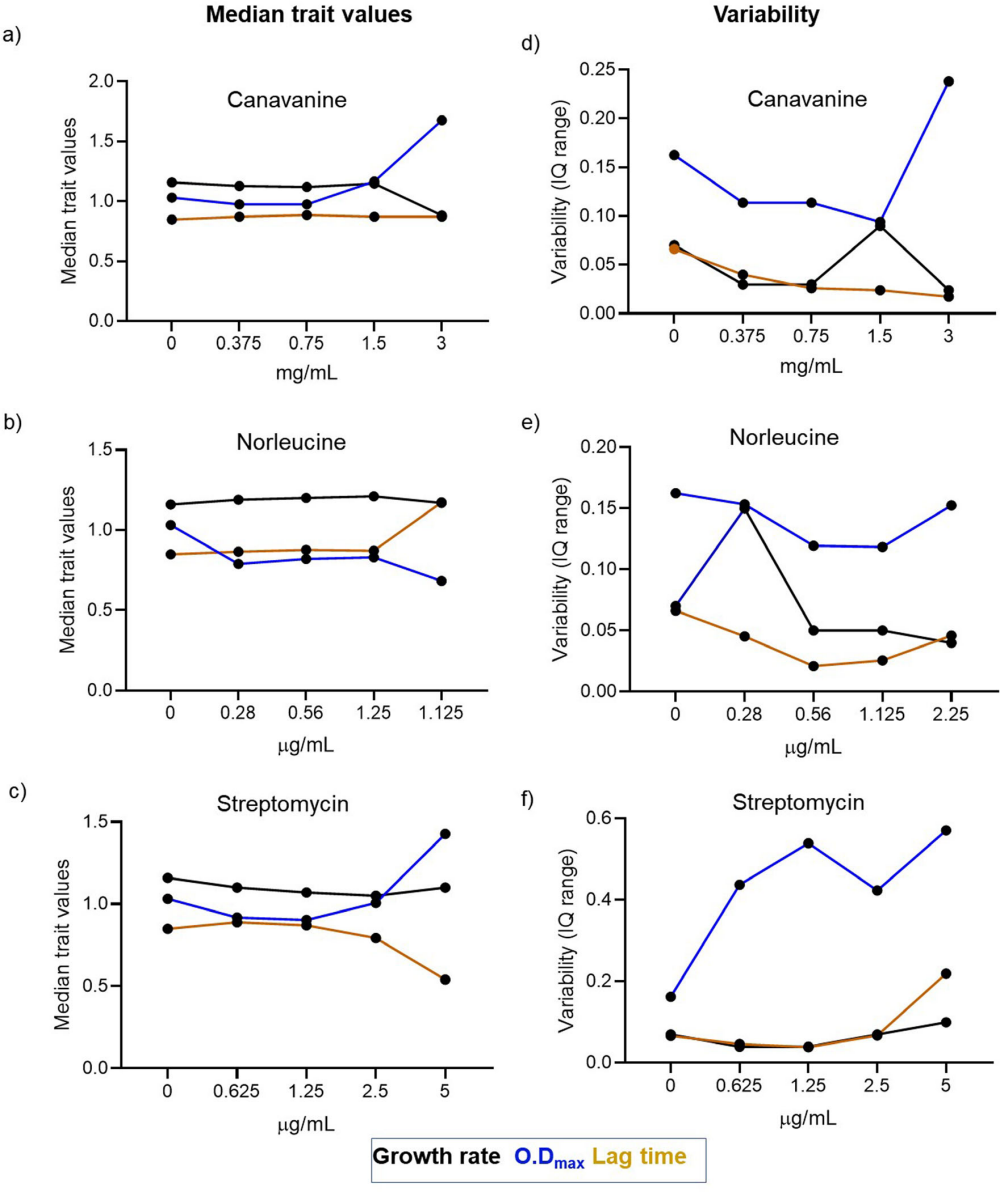
Variability in growth parameters across populations is not correlated with degree of mistranslation: (a–c) Median values for population growth rate, growth yield (OD_max600_) and lag time (time until culture reaches OD_600_ ∼0.02) as estimated from raw growth curves for WT populations treated with four concentrations of canavanine, norleucine or streptomycin (n∼40, 37 to 44 per treatment). (d–f) Variability in population growth parameters across biological replicates (n∼40, 37 to 44), estimated using the difference between the 75th and 25th percentile values for each parameter. Linear regression for variability in growth rate as a function of canavanine, norleucine or streptomycin concentration: R^2^ = 0.5, 0.3 and 0.3; for OD_max_: R^2^ = 0.8, 0.28 and 0.48; for lag time: R^2^ = 0.02, 0 and 0.6 respectively. In all panels, untreated WT is indicated as zero concentration.

While single‐cell variability clearly increased with mistranslation, population‐level variability did not show a clear correlation. To tease apart the role of mistranslation in generating population variability, we exposed WT cells to a gradient of mistranslation, by treating them with increasing concentrations of mistranslating agents (canavanine, norleucine, or streptomycin). We expected that across‐replicate variability in population growth rate, yield, and lag time (estimated using the interquartile range of each parameter) would increase monotonically with increasing mistranslation. However, we found that the degree of mistranslation was not strongly correlated with either the median trait values (Fig. 6a–c) or the variability across replicates (Fig. 6d–f). Furthermore, the patterns varied across mistranslating agents, potentially driven by the specific mode of action of each agent, or its impact on other cellular processes unrelated to mistranslation (see Discussion).

### A BRIEF BURST OF MISTRANSLATION IS SUFFICIENT TO INCREASE SUBSEQUENT POPULATION SURVIVAL

In the experiments described so far, we maintained a constant level of mistranslation throughout the course of the experiment, because mistranslation generates fresh phenotypic variability in each generation. As discussed in the Introduction, without such renewal, the impact of initial mistranslation should diminish over successive generations. However, proteome changes can be transferred across generations in other ways, such as through protein aggregates (Govers et al. 2018). We therefore asked whether a brief pulse of mistranslation can alter subsequent cell viability, and whether this effect scales with the degree of initial mistranslation, both under normal growth conditions and under stress (high temperature). We kept the window of exposure to the stress to within 2 doubling times of the slowest strain (∼2 h), so that any effects we observed were solely due to the mistranslating agent and not confounded by subsequent selection for survivors. We also ensured that the concentrations of mistranslating agents and the magnitude of the stress used did not cause any cell death within this window.

**Figure 7:**
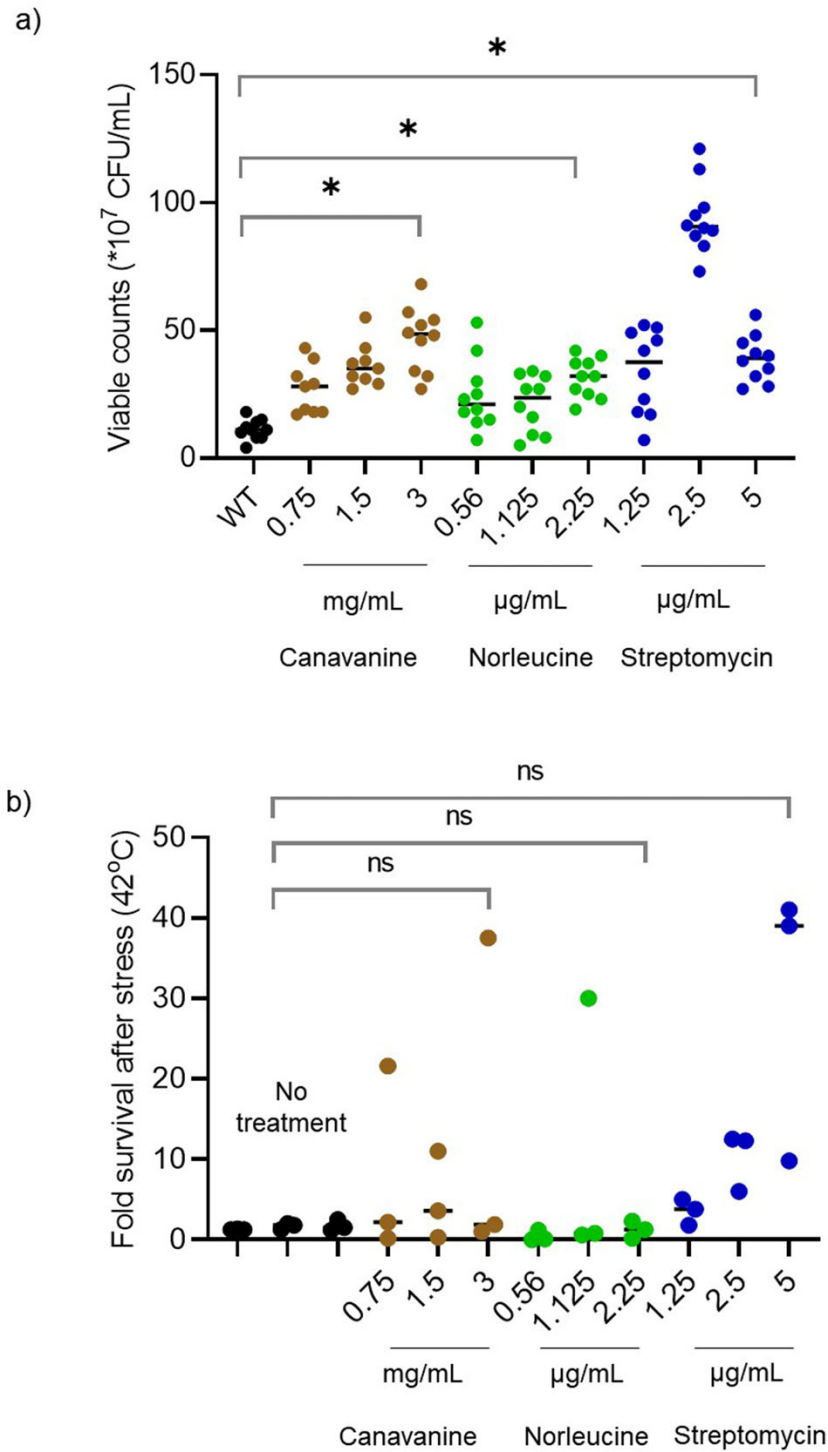
A brief burst of mistranslation enhances survival under optimal conditions. (a) Effect of treatment with different concentrations of canavanine, norleucine, or streptomycin on viable cell counts at 37°C (n = 10 per concentration). We treated log phase cultures (OD_600_∼0.6) of each strain with three concentrations of each mistranslating agent as indicated, for 2 h. Cells were then spun down, washed and dilution plated followed by incubation at 37°C for 24 h. (b) Effect of a brief exposure to different concentrations of canavanine, norleucine or streptomycin on cell survival at 42°C (n = 3 per concentration). We treated cells as above; then spun down, washed, and incubated them at 42°C for a further 2 hours in fresh medium. To calculate fold change in survival due to high temperature, we dilution plated and incubated cells at 37°C for 24 h. Horizontal bars indicate median values in both panels. Asterisks indicate significant differences in the median values.

We found that briefly exposing cells to increasing concentrations of canavanine, norleucine or streptomycin increased survival on LB agar at 37°C (Fig. 7a), consistent with our prior observations with the Mutant (Fig. 4b and Fig. 5c and d). Streptomycin was an interesting outlier, with an intermediate concentration consistently maximizing survival. We speculate that this concentration indicates a threshold beyond which the toxic effects of mistranslation overwhelm its benefits. With increasing concentrations of mistranslating agents, as before (Fig. 6a–c), we did not find a consistent trend towards higher median survival, except with streptomycin (linear regression: WT_nor_, *R* ^2^ = 0.005, *P* = 0.4; WT_can_, *R* ^2^ = 0.05, *p* = 0.18; WT_strp_, *R* ^2^ = 0.4, *p* = 0.0004). Surprisingly, at 42°C, none of the treatments significantly increased mean survival as compared with the WT (Fig. 7b). Again, survival increased with increasing concentration of streptomycin, but the other mistranslating agents did not have this effect (linear regression: WT_nor_, *R* ^2^ = 0.001, *p* = 0.9; WT_can_, *R* ^2^ = 0.03, *p* = 0.6; WT_strp_, *R* ^2^ = 0.6, *p* = 0.02). Part of the reason for the lack of difference at 42°C could be the small sample size. For logistical reasons (see Methods), we could not increase our sample size at 42°C; given the large variability across replicates, we may thus have limited power to detect a correlation.

Overall, as with population growth parameters (Fig. 6), there was no clear correlation between the extent of mistranslation and magnitude of the survival benefit at the population level. However, a brief increase in mistranslation did increase subsequent survival. In conjunction with previous results (Fig. 4b and Fig. 5c and d), these results suggest that mistranslation has the potential to influence longer‐term population and evolutionary dynamics.

## DISCUSSION

**Figure 8:**
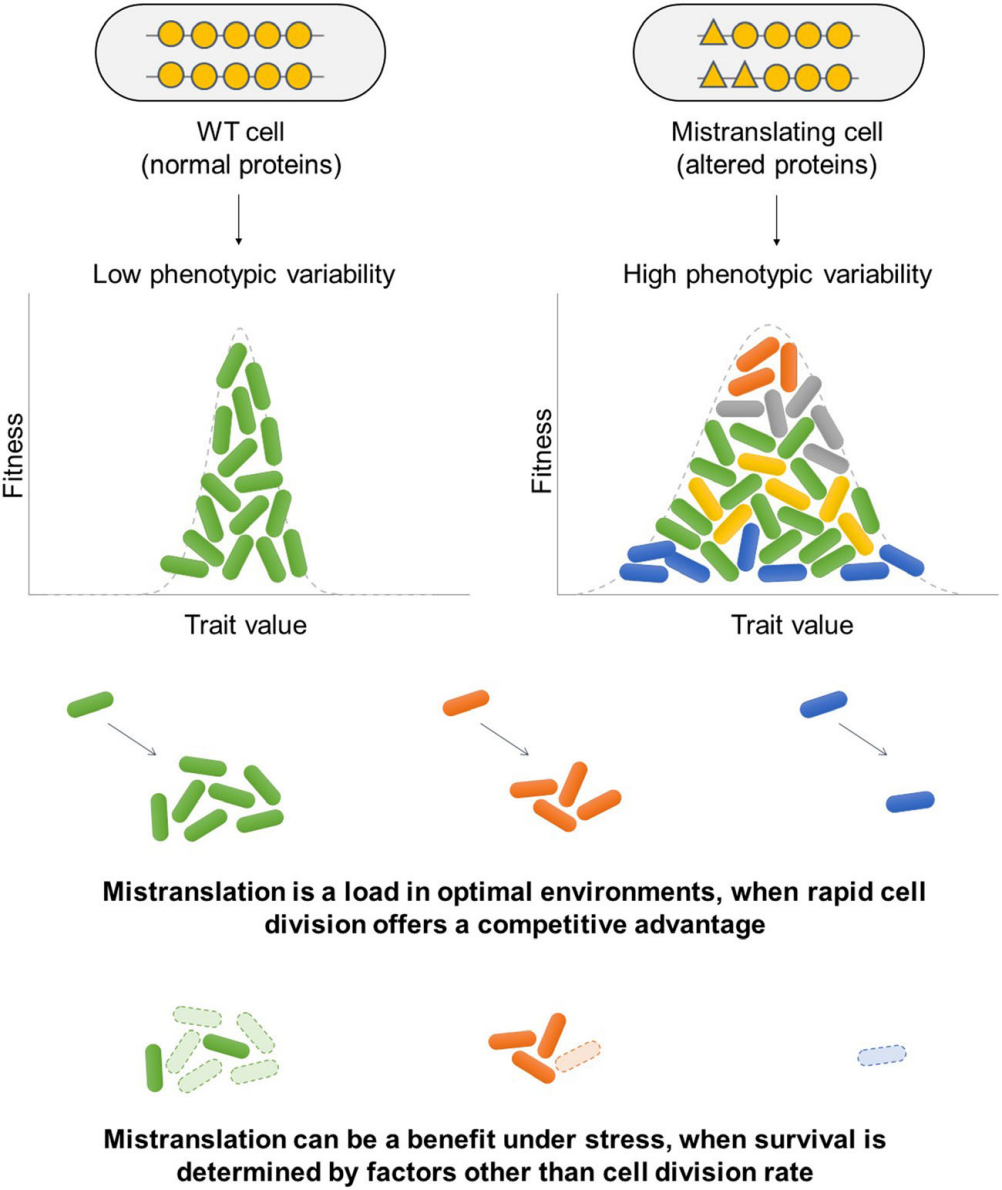
Summary of the impact of mistranslation‐induced variability on fitness.

Translational errors have been intensively studied by molecular biologists, leading to a detailed understanding of their causes and immediate cellular consequences (Kramer and Farabaugh 2007; reviewed in Ribas de Pouplana et al. 2014). At the same time, evolutionary biologists have analyzed the broader consequences of errors in cellular processes for non‐genetic adaptation (Whitehead et al. 2008; Evans et al. 2018; reviewed in Samhita 2020). However, these two perspectives have only rarely been connected, resulting in poor empirical understanding of the possible role of mistranslation for survival and adaptation in new environments. By directly manipulating mistranslation rates in multiple ways, we provide clear empirical evidence that mistranslation introduces phenotypic variability across cells; but does not consistently introduce variability across populations. Furthermore, cell‐to‐cell variability has environment‐dependent impacts on fitness: altering WT mistranslation rates in either direction is deleterious under optimal conditions, whereas mistranslation‐induced variation is associated with improved survival under stress (Fig. 8). Recently, using the same manipulations, we showed that global mistranslation increases survival under at least two stresses, DNA damage and high temperature (Samhita et al. 2020). Together, these results show that mistranslation‐induced variability has the potential to significantly alter ecological and evolutionary dynamics of populations.

Note that although mistranslation generated variability, it was difficult to establish whether the variability itself was directly responsible for growth and survival benefits; or whether these benefits arose due to secondary effects of mistranslation (see discussion below). It is also tempting to speculate that the increased variability leads to a bet hedging strategy, that is, a specific sub‐population within a heterogeneous population performs best in a new environment (for example, cells with the highest growth rates, at one end of the distribution). If so, a parent population that lacks such a sub‐population would be at a disadvantage. However, our single cell experiments do not support such a bet hedging strategy within the fitness parameters that we measured: cells that survived starvation were not drawn from a specific region of the overall distribution of cell size and time to division (Fig. 4c–d). It is still possible that mistranslating cells do sample a specific sub‐population and exhibit bet hedging, with reference to some other phenotype that we have not tested; for example, the expression level of a specific protein. Further experiments are necessary to test whether (and under what conditions) mistranslation‐induced single‐cell variability may serve as a general bet‐hedging strategy, or consistently increase population growth or survival.

Interestingly, within‐population variability did appear to generate across‐population variability in doubling time – the one growth phenotype with the same underlying trait at both the single‐cell and population levels. For the other population‐level parameters which are likely to be influenced by single cell division time (but do not measure the same trait), we found that despite the significant between‐cell variation introduced by mistranslation, population behavior largely remains repeatable (and hence predictable). What explains this discrepancy? It is possible that averaging across several single cell distributions may hide the underlying variability. For example in our case, mistranslating cells have more variable division times. If all populations consistently generate the same set of alternate proteomes (or distribution of division times), then we would not see significant variation across replicate populations. Alternatively, selection during the population growth cycle may ensure that only the fastest‐growing cells contribute to population growth rate, reducing the magnitude of variation across replicate populations. This is plausible because increased cell‐to‐cell variability in specific phenotypes (in our case, cell length and division time) also increases the number of cells with a sub‐optimal phenotype. In an unchanging or optimal environment, such maladapted cells decrease the mean trait value, leading to reduced population growth rate (Fig. 8) (Lin and Amir 2017). However, this would mean that there is some degree of correlation across successive divisions, perhaps in a phenotype other than time to division. Indeed, in fluctuating or stressful environments, both model predictions (Zhuravel et al. 2010) and prior observations (Levy et al. 2012) suggest that cell to cell variability can be beneficial, and hence likely to persist. Thus, it is possible that under fluctuating environments, cell‐to‐cell variation leads to increased variance across population fates. Further work – including modeling efforts – may help to distinguish between these possibilities, and resolve conditions under which single‐cell (between‐individual) heterogeneity should also increase between‐population variance.

Our results indicate that the underlying cause of mistranslation may play a large role in determining the impact of mistranslation‐induced variability. The effect of mistranslation varied depending on the mechanism used to increase mistranslation, as well as across different population growth parameters (doubling time, growth rate, yield, and lag time). These inconsistencies could stem from multiple factors. i) Each environmental manipulation (canavanine, norleucine, streptomycin) affects different sets of proteins. For example, canavanine and norleucine should respectively alter proteins rich in arginine and methionine; while streptomycin has a global effect, impacting all protein production. This may also explain why streptomycin tended to increase variability in all population parameters measured (Fig. 6f). The initiator tRNA depleted Mutant also causes global mistranslation, but through non‐AUG initiation, whose effects are expected to differ from that of streptomycin. Finally, hyper‐accurate ribosomes reduce the chances of codon‐anticodon mispairing, and so do not directly reverse the mistranslation caused by tRNA depletion. As such, they are not expected to cause the Mutant to revert to the WT phenotype. ii) Secondary impacts unrelated to mistranslation may complicate the relationship between the degree of mistranslation and phenotypic variability. For example, in addition to inducing mistranslation, norleucine also inhibits DNA methylation and methionine biosynthesis (Bogosian et al. 1989). Hyper‐accurate ribosomes are expected to drop off more often during translation (Karimi and Ehrenberg 1994), potentially increasing variability in the number of actively dividing cells at any given time, and altering lag time. We observed an inconsistent pattern of variability in the hyper‐accurate versus the WT and Mutant strains. Lag time across population replicates was more variable than in the parent strain both in the WT and Mutant hyper‐accurate derivatives (Fig. 3e). In addition, WT single‐cell data show tighter distributions for division time but not cell length in hyper‐accurate strains as compared with their parent strains (Fig. 2b–c, Table S1). Given the complex underlying relationships, it is perhaps not surprising that we do not see a consistent trend linking various causes of mistranslation with phenotypic variability. Thus, our work identifies the mechanistic basis of mistranslation as an important factor to understand the phenotypic effects of mistranslation.

Our work addresses gaps in prior work, broadening our understanding of the potential role of mistranslation in evolutionary dynamics. Previous studies found that mistranslation is correlated with variability in phenotype (Bacher et al. 2007; Bezerra et al. 2013) but could not establish a causal link. Others identified specific mechanisms that increased stress resistance under global mistranslation mediated by altered proteomes (Fan et al. 2015), but did not establish whether this relied on a general increase in phenotypic variability. In our work, it is reasonable to assume that a “statistical proteome” is generated in each mutant cell as a result of mistranslation (Winther and Gerdes 2011; Samhita et al. 2013). Depleting initiator tRNA content (as in our mutant) can alter the cellular proteome in various ways: via non‐AUG initiation (Winther and Gerdes 2011; Samhita et al. 2013), ribosome alterations (Shetty and Varshney 2016), or by simply lowering translation rates (Samhita et al. 2014), potentially leading to instantaneous changes in global transcript as well as protein levels. We show that completely different mechanisms of mistranslation (non‐AUG initiation accompanied by decreased translation; replacing an amino acid with a non‐native analog; increasing decoding errors) all converge on the pattern of increased variability in growth and survival. Thus, the potentially varied and specific mechanisms linking various forms of mistranslation to phenotypic variation ultimately achieve the same endpoint under stress: an increased probability of population survival. In addition, compared to prior work, our results are somewhat more applicable to *E. coli* function and evolution in natural ecosystems. For instance, the ecological relevance of genetic manipulations used in prior work (such as mutations in the ribosomal protein S4 (Fan et al. 2015; Bratulic et al. 2017)) is unclear. In contrast, translation – and specifically translation initiation – is reduced in response to several environmental stresses (Nagase et al. 1988; Winther and Gerdes 2011; Watanabe et al. 2013), creating a cellular environment analogous to that of our mistranslating mutant with reduced initiator tRNA. Second, we used two stressful conditions – starvation and high temperature – that lead to increased cell death in *E.coli* and are thought to be encountered by *E.coli* in its natural habitat (reviewed in Koch 1971; van Elsas et al. 2011). Finally, we measured the impact of mistranslation on cell growth and survival, which are key parameters governing microbial ecological and evolutionary dynamics and have significant repercussions for genome structure and evolution (Roller et al. 2016). Thus, we speculate that cells could modulate mistranslation levels as a generalized, global response to multiple stressful conditions that they encounter in nature.

In closing, we note that our results lead to a number of interesting open questions. For instance, while we observe that mistranslation reliably generates variability, we do not know if the same variants are re‐generated across generations; precisely which variants survive under each stressful condition; and whether this is predictable across environments. More work is also needed to clarify whether advantageous variants pave the way for the phenotype to be fixed by mutation, as suggested previously (Cowen and Lindquist 2005; Whitehead et al. 2008). Finally, while our experiments inform about the short‐term impact of altering mistranslation, the longer‐term impacts of mistranslation need to be investigated further. For instance, recent work showed that a mistranslating strain fixes distinct sets of beneficial mutations during laboratory adaptation to antibiotic stress (Bratulic et al. 2017). It would be exciting if these results could be generalized across various stresses and forms of mistranslation. Here, we have built a foundation to address these questions by demonstrating that mistranslation can influence short‐term population trajectories and set the stage for longer‐term evolutionary consequences.

## MATERIALS AND METHODS

### BACTERIAL STRAINS

To manipulate mistranslation levels in wild type (WT) KL16 *E. coli* cells (Low 1968), we used two genetically altered derivatives of the WT. As our focal ‘mistranslating’ strain, we used the KL*ΔZWV* strain (henceforth ‘mutant’), which lacks three of the four initiator tRNA genes encoded by *E. coli* (Samhita et al. 2013). Initiator tRNA acts only at the first step of protein synthesis and has no substitute (Gualerzi and Pon 2015). Since the mutant carries ∼25% of the WT initiator tRNA complement, it has a lower rate of protein synthesis, a ∼20% slower growth rate than the WT (Samhita et al. 2020), and mistranslates through non‐AUG initiation (Samhita et al. 2013). In contrast, to reduce mistranslation rates in the WT, we introduced a mutation (K42R) in the protein S12 that increases translation accuracy by reducing the frequency of decoding errors (Chumpolkulwong et al. 2004). We transferred this mutation into KL16 and KL*ΔZWV* from the parent strain SS3242 obtained from CGSC, Yale University, via P1 transduction, generating strains KL(HA) and KL*ΔZWV*(HA), referred to as WT(HA) and Mutant(HA) in the text. The mutation led to an ∼10‐fold increase in translation accuracy both in the WT and in the Mutant (Samhita et al. 2020). Note that the introduction of hyper‐accurate ribosomes will not reverse the non‐AUG initiation seen in the Mutant strain; however, it lowers overall background mistranslation levels by reducing decoding errors, as mentioned above. For single‐cell variability measurements, we used WT and mutant strains carrying a genomically encoded, constitutively expressed GFPmut2 allele tagged with a kanamycin resistance marker inserted between the genes *aidB* and *yjfN*, and expressed from a P5 promoter (original strain gifted by Prof Bianca Sclavi, ENS, Paris). All strains and genotypes are listed in Table 1.

### GROWTH CONDITIONS AND MEDIA

When generating strains or to simulate control (normal growth) conditions, we grew bacterial cultures in Luria Bertani medium (LB) or on LB‐agar plates containing 1.8% (w/v) agar (Difco), incubated at 37°C. In some experiments, we also altered growth conditions to elevate mistranslation levels and/or impose stress, as follows. To increase mistranslation levels, we added (1) canavanine sulphate at concentrations ranging from to 0.375 to 3 mg/mL as specified in each experiment (canavanine is an analogue of arginine that induces mistranslation) (Fan et al. 2015), (2) norleucine (an analog of leucine which substitutes for the amino acid methionine in proteins (Karkhanis et al. 2007)) at concentrations ranging from 0.28 to 2.25 μg/mL and (3) streptomycin sulphate at concentrations ranging from 0.625 to 5 μg/mL (streptomycin leads to errors in ribosomal decoding (Carter et al. 2000)). To impose stress, we subjected single cells to starvation by supplying only saline instead of a growth medium (i.e. no nutrients, for measures of survival). At the population level, we let cultures grow until late stationary phase when nutrients are depleted (which imposes stress on the cells (Koch 1971)) and then carried out competition experiments. Finally, we cultured cells in LB at high temperature (42°C), imposing stress that reduces survival (van Elsas et al. 2011). To check the impact of a transient increase in mistranslation on survival at 42°C, we exposed replicate cultures to different concentrations of canavanine, norleucine or streptomycin for ∼2 h until they reached OD_600_ ∼0.6. We then pelleted and re‐suspended cells and cultured them for 2 h at 42°C before carrying out dilution plating to assess survival. Each treatment had triplicates before and after exposure to 42°C, i.e. a total of 6 cultures. Given three concentrations and three mistranslating agents, this led to 18*3 = 54 agar plates, which were done in staggered sets of 18 each. Handling more than 18 at a time led to significant time variation in the steps of cell pelleting and exposure to 42°C, adding to the variability that we were attempting to capture. As a result, at any given time, we were limited to three treatments for a given mistranslating agent. In addition, we could not compare across the three blocks of the same experiment because of the high across‐experiment variability in growth and colony numbers.

### SINGLE CELL MICROFLUIDICS MEASUREMENTS

To measure variability in growth characteristics at the single cell level, we used a microfluidic device seeded with GFP‐labelled *E. coli* strains (described above). The ‘mother machine’ microfluidic device was fabricated as previously described (Wang et al. 2010). Saturated overnight cultures of WT, Mutant, WT(HA), and Mutant(HA) were sub‐cultured to 1% by volume into LB and incubated at 37^°^C for 3 h. To concentrate cells ∼20 fold, we centrifuged the cultures (5 min, 3000g) and re‐suspended cells in 200 μL LB. We injected the cell suspension into the microfluidic device using a syringe, and allowed cells to diffuse into the growth channels (∼ 2 h; see schematic in Fig. 4a). Then, the device was placed in a temperature‐controlled stage‐top incubator (OKOlab), which in turn was placed on an inverted microscope (Olympus IX81). To allow cell growth in the device, we pumped LB from a 15 mL centrifuge tube (Greiner) held at a constant temperature in a dry block heater (IKA). We allowed cells to grow in the device for 2.5 h at a media flow rate between 600 – 800 μL/h, and then began imaging cells to measure growth characteristics. When necessary, saline was used in place of LB to simulate starvation (since cells cannot divide in saline but will remain osmotically intact). We used a coolLED lamp (excitation: 490 nm) for fluorophore excitation, and captured bright field and fluorescence images at intervals of 2 min for 10 h at 40X magnification using an EMCCD camera (Photometrics Prime). We imaged ∼160 channels at constant flow and temperature. We then carried out preliminary image editing using ImageJ and a custom MATLAB code (MathWorks), and extracted information on cell length and division time, by binarizing the images and assigning an identity to each cell (Banerjee et al. 2020). Based on changes in fluorescence intensity in the cell body, the code identified a cell division (see Fig. S12). We measured cell length at every frame to calculate cell length at birth and division, and the corresponding time for each event. For all experiments, we collected data in two or three independent blocks (each block conducted on a different day, with independent starting cultures). However, for two of the experimental blocks at 37°C, the actual temperature (recorded by the instrument during the experiment) was 35°C or 39°C; hence, we report the results of each block separately. At 42°C, we could only conduct a single experimental block. When counting live and dead cells under stress (3 experimental blocks), an instantaneous loss of fluorescence signal was used as an indicator of cell death.

### BOOTSTRAP AND CONVERGENCE ANALYSIS

Since our single‐cell phenotype distributions were non‐parametric, accurately estimating and comparing mean and variance of the distributions was challenging. Hence, we used bootstrap analysis to estimate the error in the mean *X* of each dataset as follows (Efron and Tibshirani 1994): for a dataset *D* = (Samhita 2020) with *N* entries, a large number of realizations of the dataset is generated by randomly sampling entries with replacement. A given realisation is thus produced by picking *N* randomly selected entries from the dataset; and a given entry may be picked multiple times. This procedure is repeated many times, producing many realizations. For each realization, the mean and variance are calculated, resulting in a distribution of the realization estimators. The error in the estimators is then given by the standard deviation of the distribution. For the analysis shown in this paper, we implemented bootstrapping using the standard MATLAB (v2019a) function ‘bootstrap’. We first tested for convergence in parameter estimates using a range of sample sizes (*N* values; Fig. S4); and then used the largest *N* (10^6^) to estimate the mean and variance in the distribution of each phenotype of interest (e.g. cell length or time to division), for each strain or treatment.

### MEASURING POPULATION GROWTH AND YIELD

To measure variability in growth across populations, we used 40 independent colonies of each *E. coli* strain as biological replicates. We inoculated colonies in LB and allowed them to grow overnight at 37°C with shaking at 200 rpm for 16 hours. We then added 5 μL of the overnight culture into 495 μL of the relevant growth medium in 48 well microplates (Corning‐Costar), and incubated them in a shaking tower (Liconic) at 37°C. We measured optical density (OD) of each well at 600 nm using an automated growth measurement system (Tecan, Austria), every 30 or 40 minutes for 12 to 18 hours. The automated system allowed us to simultaneously measure growth rates in up to 10 microplates. We estimated maximum growth rate using the Curve Fitter software (Delaney et al. 2013) and maximum OD value (OD_max_, as a proxy for growth yield) by averaging the five highest OD values. Doubling time was estimated as ln2/maximum growth rate. Lag time was measured as the time taken until cultures reached OD_600_ ∼0.02.

### MEASURING CELL SURVIVAL

We measured cell survival in liquid culture by plating serial dilutions of the culture on agar medium and counting colonies (which represent viable cells from the original culture). Briefly, we set up 20 replicate cultures of a strain (each inoculated from an individual colony) and allowed cultures to grow to saturation overnight in rich media (LB) or under stress, as required. We then sub‐cultured cells 1% by volume and set up the experiment. At appropriate time intervals, we serially diluted the culture (55 μL of the focal culture diluted it in 495 μL of normal saline) until we obtained sufficiently dilute cultures such that the final dilution plated on LB agar would give rise to ∼20 to 200 distinct colonies (which can be counted reliably). We incubated plates for 24 hours, counted colonies, and multiplied by the appropriate dilution factor to determine viable counts in the original culture.

### MEASURING COMPETITIVE FITNESS

To test the relative fitness of WT and mutant strains under competition, we first had to establish a way to distinguish the two strains. To do so, we generated KL*ΔlacZ* (WT lacking the *lacZ* gene). This strain forms white colonies on MacConkey's agar, while the other strains under study – Mutant, WT(HA) and Mutant(HA) – form pink colonies because they carry an intact *lacZ* gene. We grew the two strains being competed to saturation (overnight) independently and then mixed them (1% each by volume) into 5 mL of growth medium. We then subjected the mix to periodic dilution plating onto MacConkey's agar, and determined the relative numbers of each strain over time. We confirmed that the *lacZ* deletion is selectively neutral, by competing it against the unmarked parent WT strain (Fig. S1).

### QUANTIFYING VARIABILITY

As described above, we collected data on the size, length, and division time of single cells; and parameters such as growth rate, growth yield (OD_max_) and lag time for replicate populations of each strain or experimental treatment. In most cases, the data were not distributed normally (Shapiro Wilke test for normality). Therefore, we could not use standard quantifications of variability such as the variance or coefficient of variation (CV = standard deviation/mean). To assess differences in variability across groups, we employed a non‐parametric statistical test‐ Fligner‐Killeen test‐ that is robust to departures from normality (Fligner and Killeen 1976; Conover et al. 1981). This test ranks all data around the median value, measures the values of the residuals for each data point, and calculates the test statistic by ranking the residual values. All comparisons are tabulated in Table S1. As appropriate, we visualized variability using the frequency distribution of each measured variable, or the interquartile range of the data.

## AUTHOR CONTRIBUTIONS

L.S. and D.A. conceived the project; L.S., D.A., and S.T. designed experiments; L.S., P.R., and G.S. conducted experiments; L.S., G.S., S.T., and D.A. analyzed data; L.S. and D.A. wrote the manuscript with input from all authors.

## CONFLICT OF INTEREST

The authors declare no conflict of interest.

## DATA ARCHIVING

https://doi.org/10.5061/dryad.612jm642m

Associate Editor: C. Burch

Handling Editor: T. Chapman

## Supporting information

Figure S1. lacZ deletion does not influence the outcome of pair‐wise competition experiments: Log phase cultures (OD600∼0.6) of WT and WTΔlacZ were subjected to pair‐wise competition experiments in LB at 37°C followed by plating on MacConkey's agar. Percentage fraction of each strain is plotted against time.
Figure S2: Probability density function of cell length and division time of single cells as monitored in the microfluidics device.
Figure S3. Distributions of time to division for individual mother cell divisions: Individual mother cells (n=3) from WT and Mutant were monitored in the microfluidics device for ∼60 cell divisions each, at 37°C.
Figure S4. Parameter convergence in bootstrap analysis: Left panels: Illustration of convergence in estimated parameter values (mean and variance in single‐cell phenotype) as a function of number of bootstrap samples, for WT (black) and Mutant (red) at 32°C.
Figure S5. Raw growth curves showing independent biological replicates: Raw growth curves for the strains indicated (n=37 to 44) showing OD600 over time plots for each biological replicate (corresponding to a single colony) as obtained by a Tecan growth reader recording OD600 every 30 minutes.
Figure S6. Mistranslation does not impact variability in growth rate across replicate populations: Violin plots showing the distributions of growth rates estimated using ∼40 (37 to 44) biological replicates (populations) for each strain or growth condition. Median, 25th and 75th quartiles are indicated by solid lines within each violin.
Figure S7. Mistranslation impacts mean doubling time across replicate populations: Violin plots showing distributions of doubling times estimated using ∼40 (37 to 44) biological replicates (populations) for each strain or growth condition.
Figure S8. Mistranslation impacts mean fitness across replicate populations: Violin plots showing distributions of three population growth parameters, estimated using ∼40 (37 to 44) biological replicates (populations) for each strain or growth condition.
Figure S9. Results of bootstrap analyses of single cell phenotype at 42 °C: (a) Left panels: convergence in parameter estimates as a function of number of bootstrap samples.
Figure S10. Cell death under starvation stress: Number of WT and Mutant cells that either lived or died in microfluidic channels during starvation conditions, in 3 independent experimental blocks (Sets 1, 2 or 3).
Figure S11. Replicate blocks for pair‐wise competition experiments: Pair‐wise growth competition experiments, strains and conditions as indicated, see Figure 4b–e.
Figure S12: WT and Mutant cells within a representative single growth channel, with the fluorescence intensity plotted from the region of interest drawn across the cells.

Table S1: Statistical comparisons of data
