# Supplementary material for: The impact of mistranslation on phenotypic variability and fitness
Source: Evolution. 2021 Feb 2;75(5):1201–17. doi: 10.1111/evo.14179 (PMC8248024; doi:10.1111/evo.14179)
Supplement: Supplementary file 1 — Figure S1. lacZ deletion does not influence the outcome of pair‐wise competition experiments: Log phase cultures (OD600∼0.6) of WT and WTΔlacZ were subjected to pair‐wise competition experiments in LB at 37°C followed by plating on MacConkey's agar. Percentage fraction of each strain is plotted against time. Figure S2: Probability density function of cell length and division time of single cells as monitored in the microfluidics device. Figure S3. Distributions of time to division for individual mother cell divisions: Individual mother cells (n=3) from WT and Mutant were monitored in the microfluidics device for ∼60 cell divisions each, at 37°C. Figure S4. Parameter convergence in bootstrap analysis: Left panels: Illustration of convergence in estimated parameter values (mean and variance in single‐cell phenotype) as a function of number of bootstrap samples, for WT (black) and Mutant (red) at 32°C. Figure S5. Raw growth curves showing independent biological replicates: Raw growth curves for the strains indicated (n=37 to 44) showing OD600 over time plots for each biological replicate (corresponding to a single colony) as obtained by a Tecan growth reader recording OD600 every 30 minutes. Figure S6. Mistranslation does not impact variability in growth rate across replicate populations: Violin plots showing the distributions of growth rates estimated using ∼40 (37 to 44) biological replicates (populations) for each strain or growth condition. Median, 25th and 75th quartiles are indicated by solid lines within each violin. Figure S7. Mistranslation impacts mean doubling time across replicate populations: Violin plots showing distributions of doubling times estimated using ∼40 (37 to 44) biological replicates (populations) for each strain or growth condition. Figure S8. Mistranslation impacts mean fitness across replicate populations: Violin plots showing distributions of three population growth parameters, estimated using ∼40 (37 to 44) biological replicates (populati [file EVO-75-1201-s002.pdf]

Figure S1

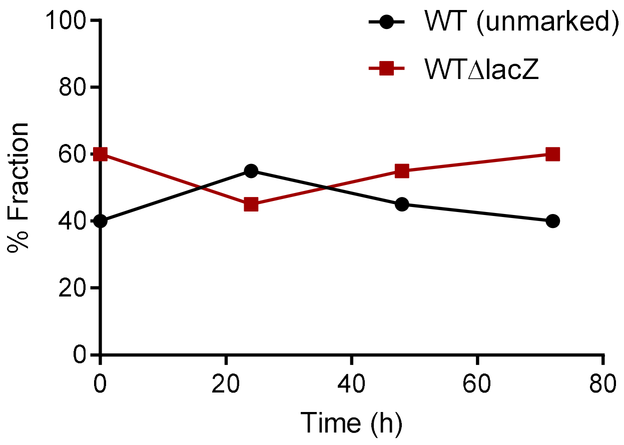

Figure S1. *lacZ* deletion does not influence the outcome of pair-wise competition experiments: Log phase cultures (OD_{600}~0.6) of WT and WTΔ*lacZ* were subjected to pair-wise competition experiments in LB at 37°C followed by plating on MacConkey's agar. Percentage fraction of each strain is plotted against time.

Figure S2

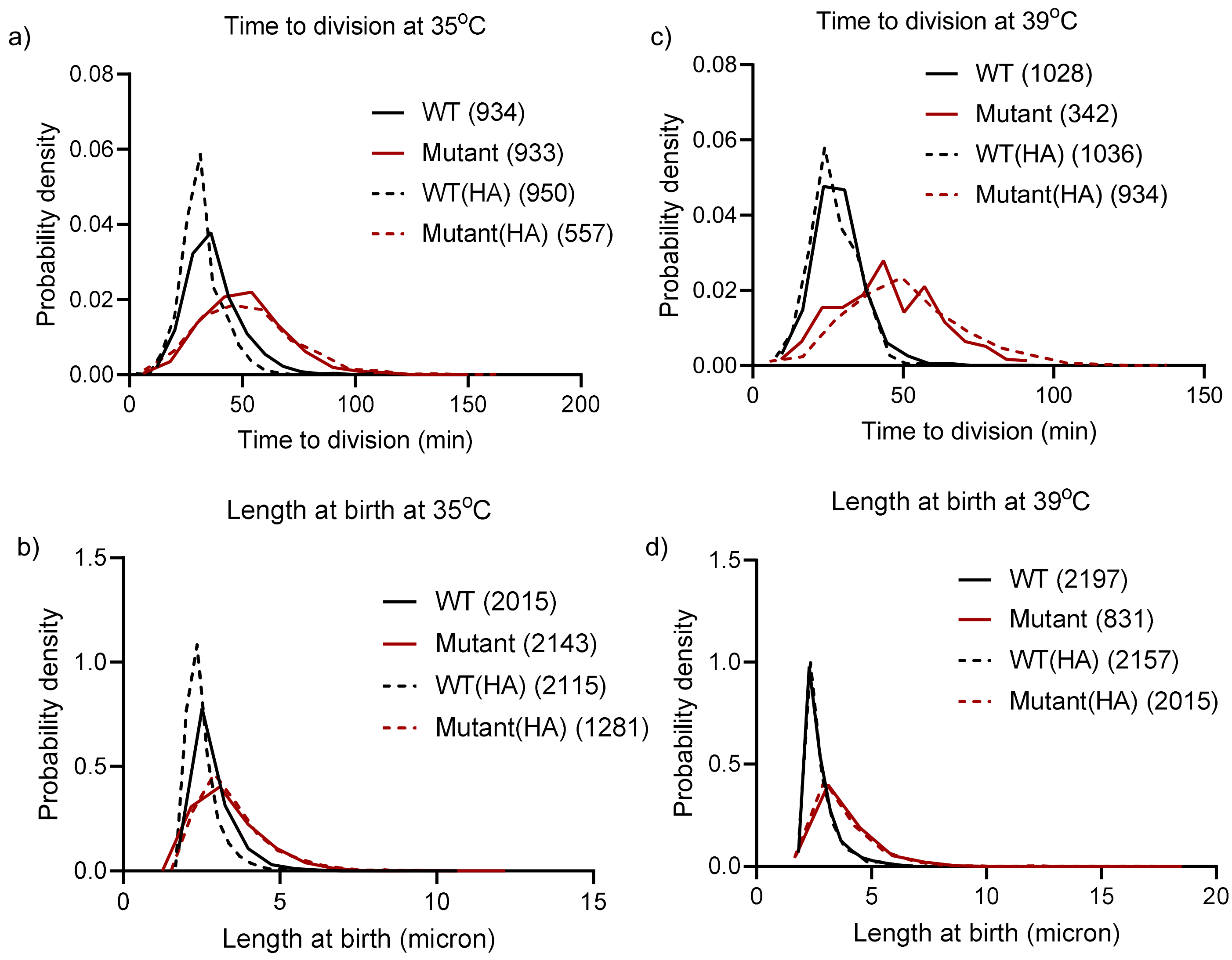

Figure S2: Probability density function of cell length and division time of single cells as monitored in the microfluidics device. Wider distributions indicate greater cell-to-cell variation. Total number of cells (n) is indicated within parentheses in the key. WT=wild type; HA=hyper-accurate strains of either WT or Mutant.

Figure S3

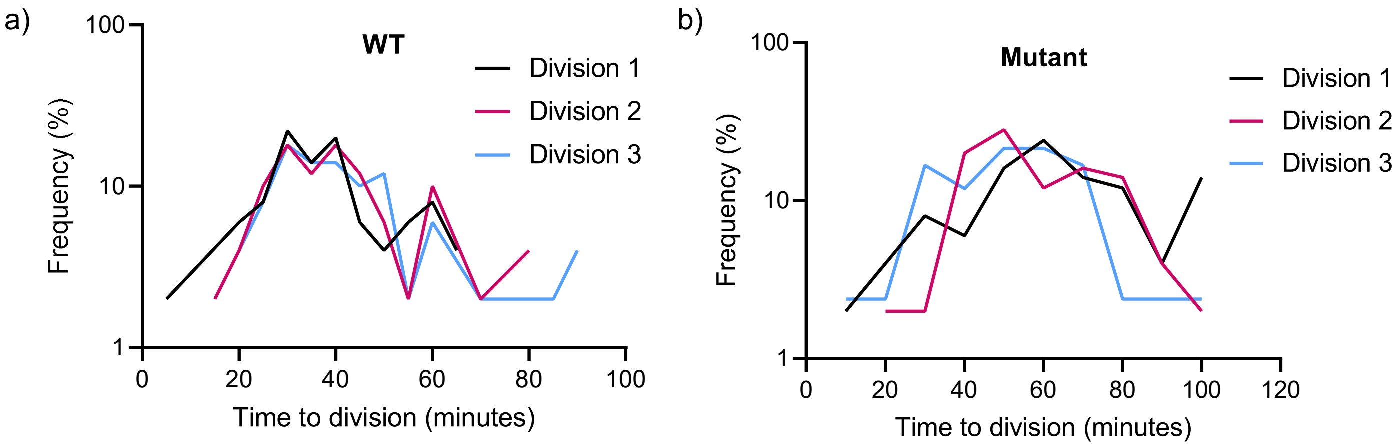

Figure S3. Distributions of time to division for individual mother cell divisions: Individual mother cells (n=3) from WT and Mutant were monitored in the microfluidics device for ~60 cell divisions each, at 37°C. Frequency distributions arising from each such lineage of divisions is plotted here against time to division. For WT, comparison of means across the three distributions, Welch's ANOVA test, W=1.9, P=0.15. For Mutant, comparison of means across the three distributions, Welch's ANOVA test, W=4.3, P=0.02

Figure S4

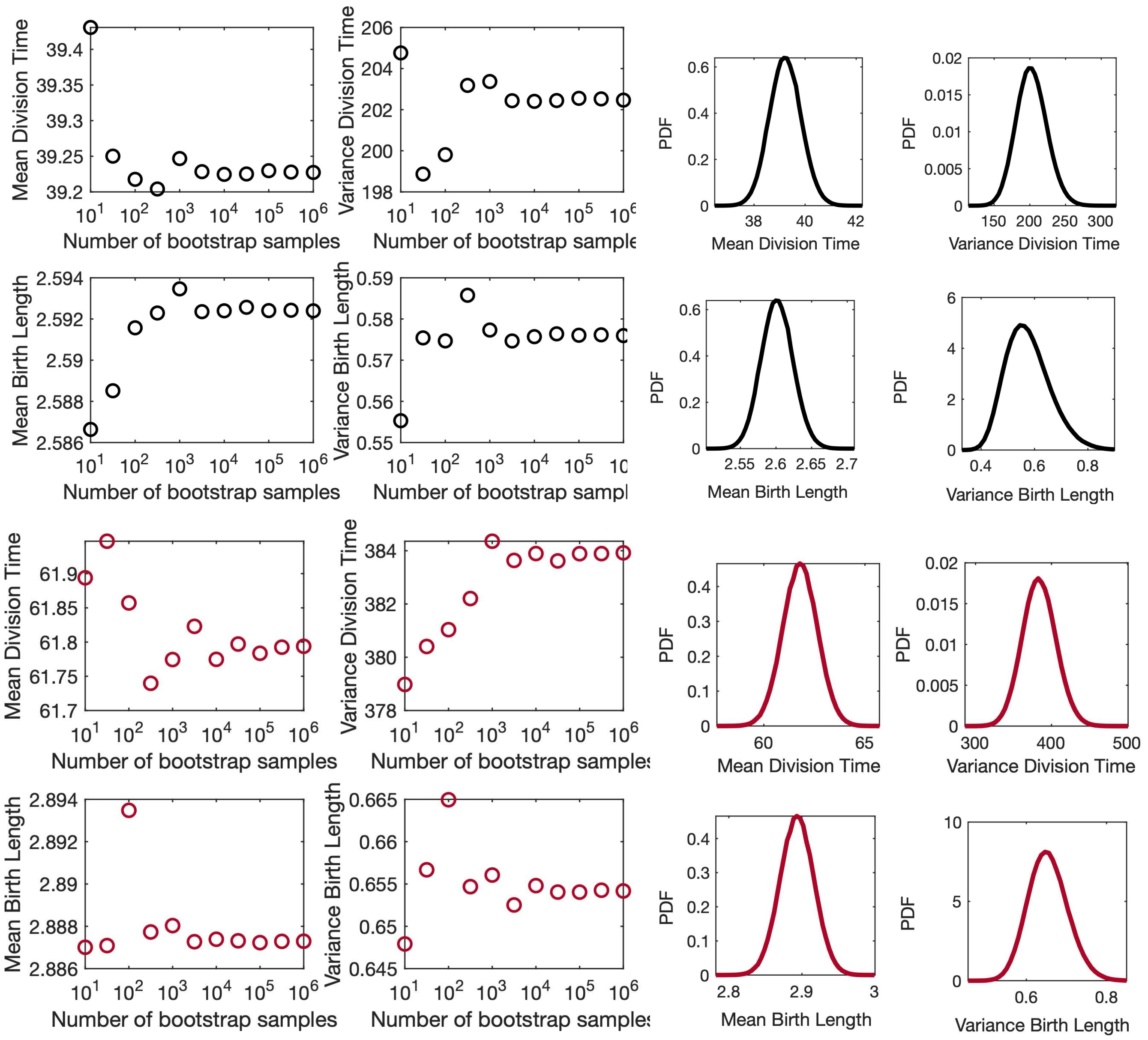

Figure S4. Parameter convergence in bootstrap analysis: Left panels: Illustration of convergence in estimated parameter values (mean and variance in single-cell phenotype) as a function of number of bootstrap samples, for WT (black) and Mutant (red) at 32ºC. Right panels: Distributions of estimated parameter values (mean and variance) are well characterised by a Gaussian distribution at the highest bootstrap sample size (1 million). We used these distributions to calculate the mean and variance, shown in Fig 2.

Figure S5

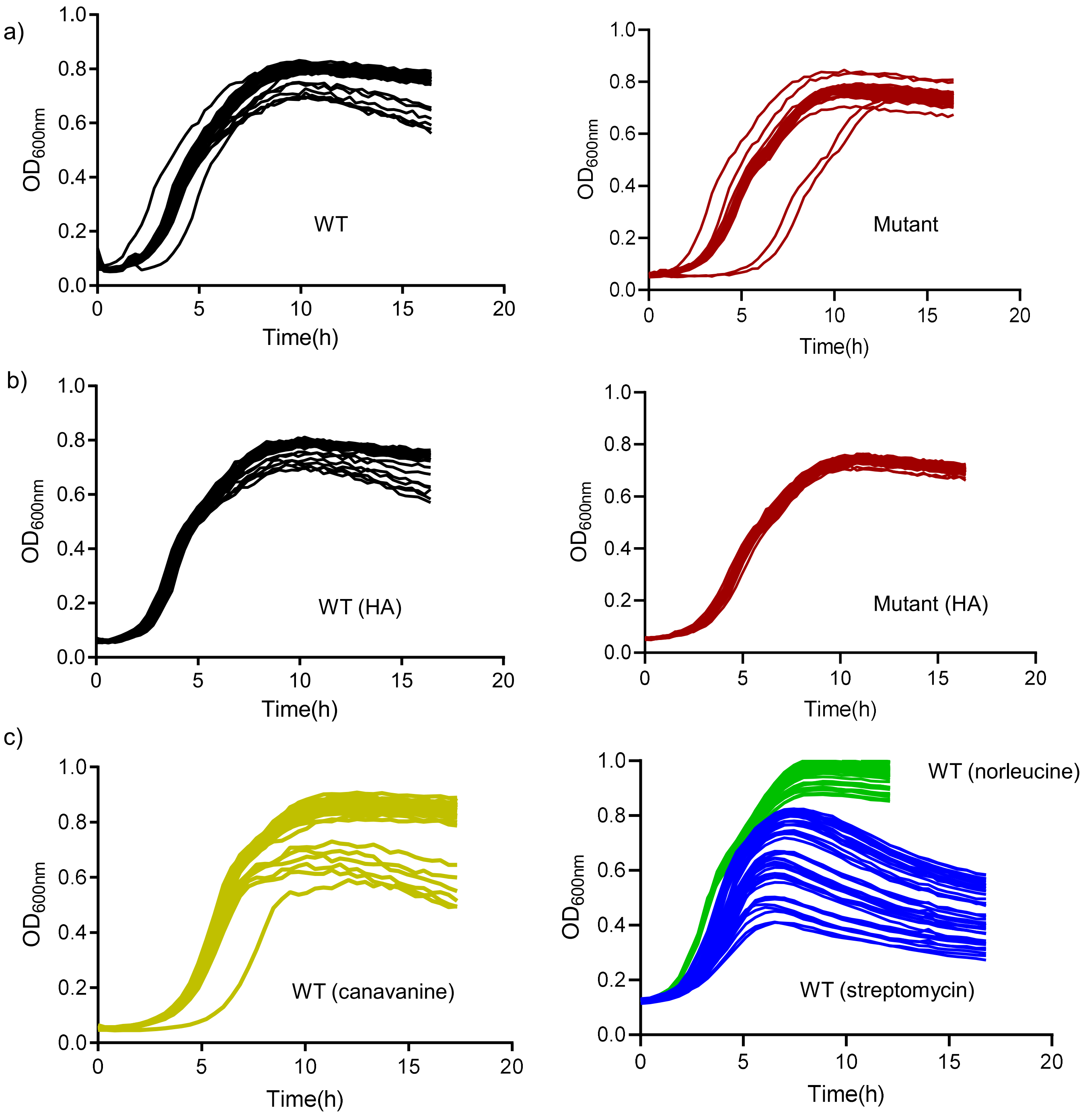

Figure S5. Raw growth curves showing independent biological replicates: Raw growth curves for the strains indicated (n=37 to 44) showing OD_{600} over time plots for each biological replicate (corresponding to a single colony) as obtained by a Tecan growth reader recording OD_{600} every 30 minutes. Canavanine (3 mg/mL), Norleucine (2.25 μg/mL) and Streptomycin (5 μg/mL) were added to the growth medium of LB where indicated.

Figure S6

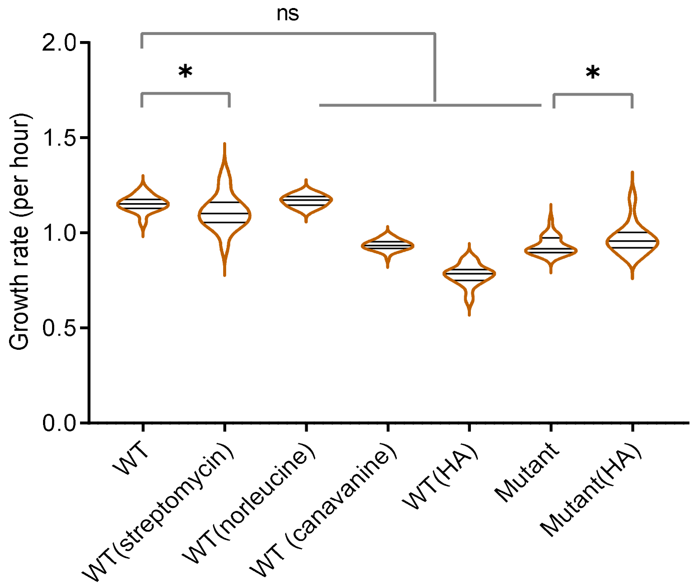

Figure S6. Mistranslation does not impact variability in growth rate across replicate populations: Violin plots showing the distributions of growth rates estimated using ~40 (37 to 44) biological replicates (populations) for each strain or growth condition. Median, 25th and 75th quartiles are indicated by solid lines within each violin. The length of each violin corresponds to the range of the distribution. Asterisks indicate significant differences in variability. WT=wild type; HA=hyper-accurate.

Figure S7

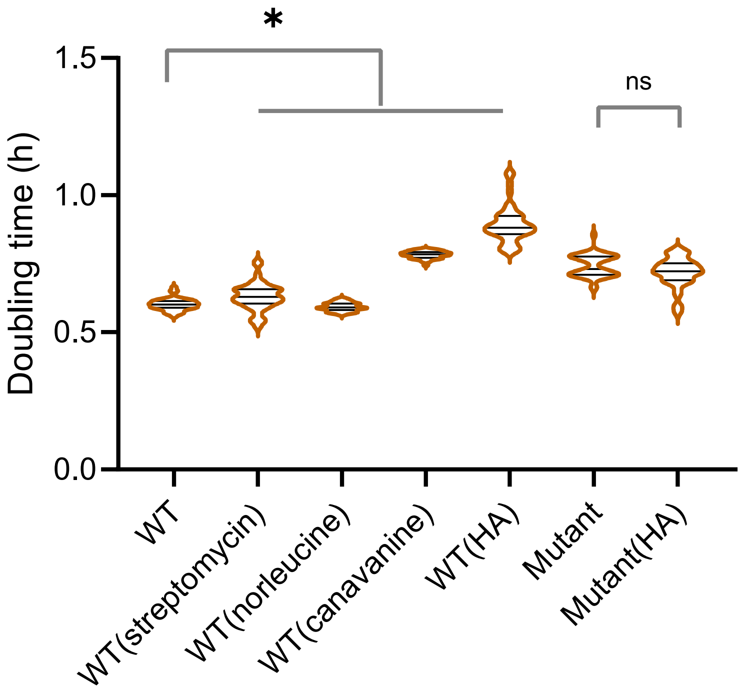

Figure S7. Mistranslation impacts mean doubling time across replicate populations: Violin plots showing distributions of doubling times estimated using ~40 (37 to 44) biological replicates (populations) for each strain or growth condition. Median, 25th and 75th quartiles are indicated by solid lines within each violin. The length of each violin corresponds to the range of the distribution. Asterisks indicate significant differences in median values. WT=wild type; HA=hyper-accurate.

Figure S8

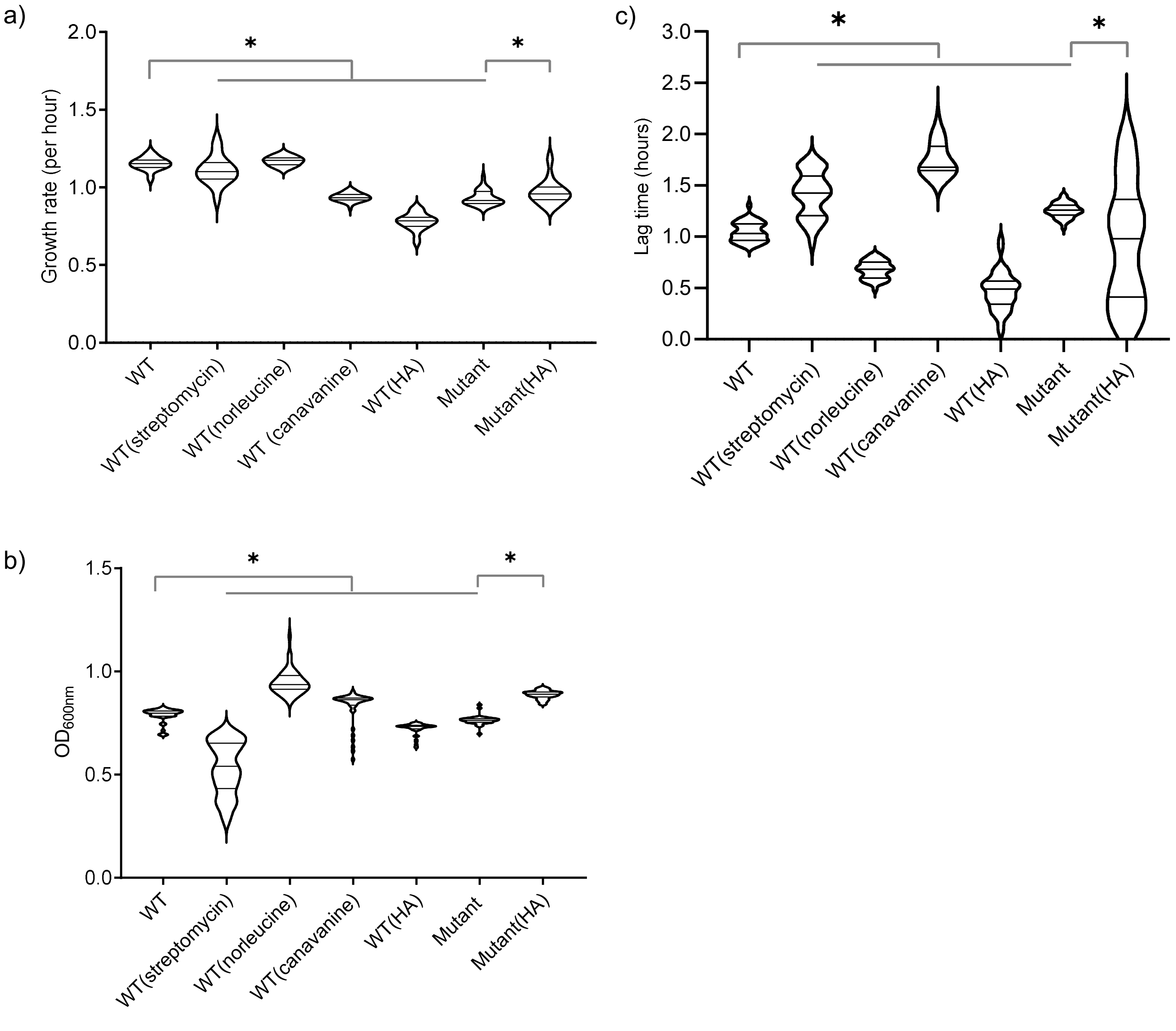

Figure S8. Mistranslation impacts mean fitness across replicate populations: Violin plots showing distributions of three population growth parameters, estimated using ~40 (37 to 44) biological replicates (populations) for each strain or growth condition. (a) Growth rate (b) growth yield and () lag time (time until culture reaches OD_{600} ~0.02). Median, 25th and 75th quartiles are indicated by solid lines within each violin. The length of each violin corresponds to the range of the distribution. Asterisks indicate significant differences in median values. WT=wild type; HA=hyper-accurate.

Figure S9

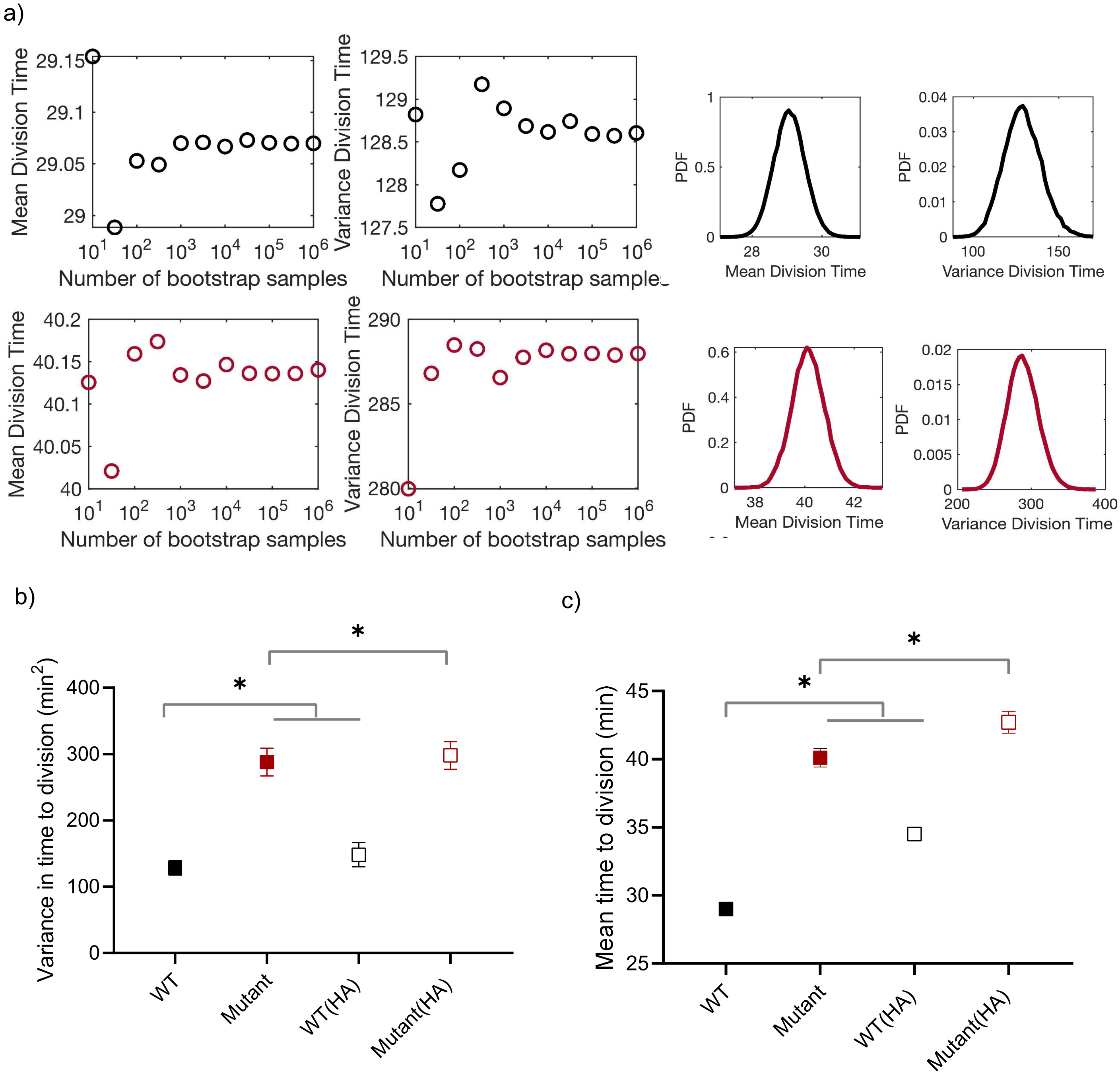

Figure S9. Results of bootstrap analyses of single cell phenotype at 42°C: (a) Left panels: convergence in parameter estimates as a function of number of bootstrap samples. Examples for two data sets are shown here (Black=WT, Red=Mutant); right panels: distributions for parameter values at the largest sample size (1 million) are well characterised by a Gaussian distribution, which are reported in the main text and panel b. (b) Plots showing the estimated mean and variance in time to division of WT, Mutant and hyper-accurate strains at 42°C from 10^6 bootstrap samples. Asterisks indicate significant pairwise differences based on Tukey's HSD.

Figure S10

	Set 1			Set 2			Set 3		
	Live	Dead	% Dead	Live	Dead	% Dead	Live	Dead	% Dead
WT	160	37	18.8	286	73	20.3	329	69	17.3
Mutant	197	10	4.8	409	19	4.4	285	9	3

Figure S10. Cell death under starvation stress: Number of WT and Mutant cells that either lived or died in microfluidic channels during starvation conditions, in 3 independent experimental blocks (Sets 1, 2 or 3).

Figure S11

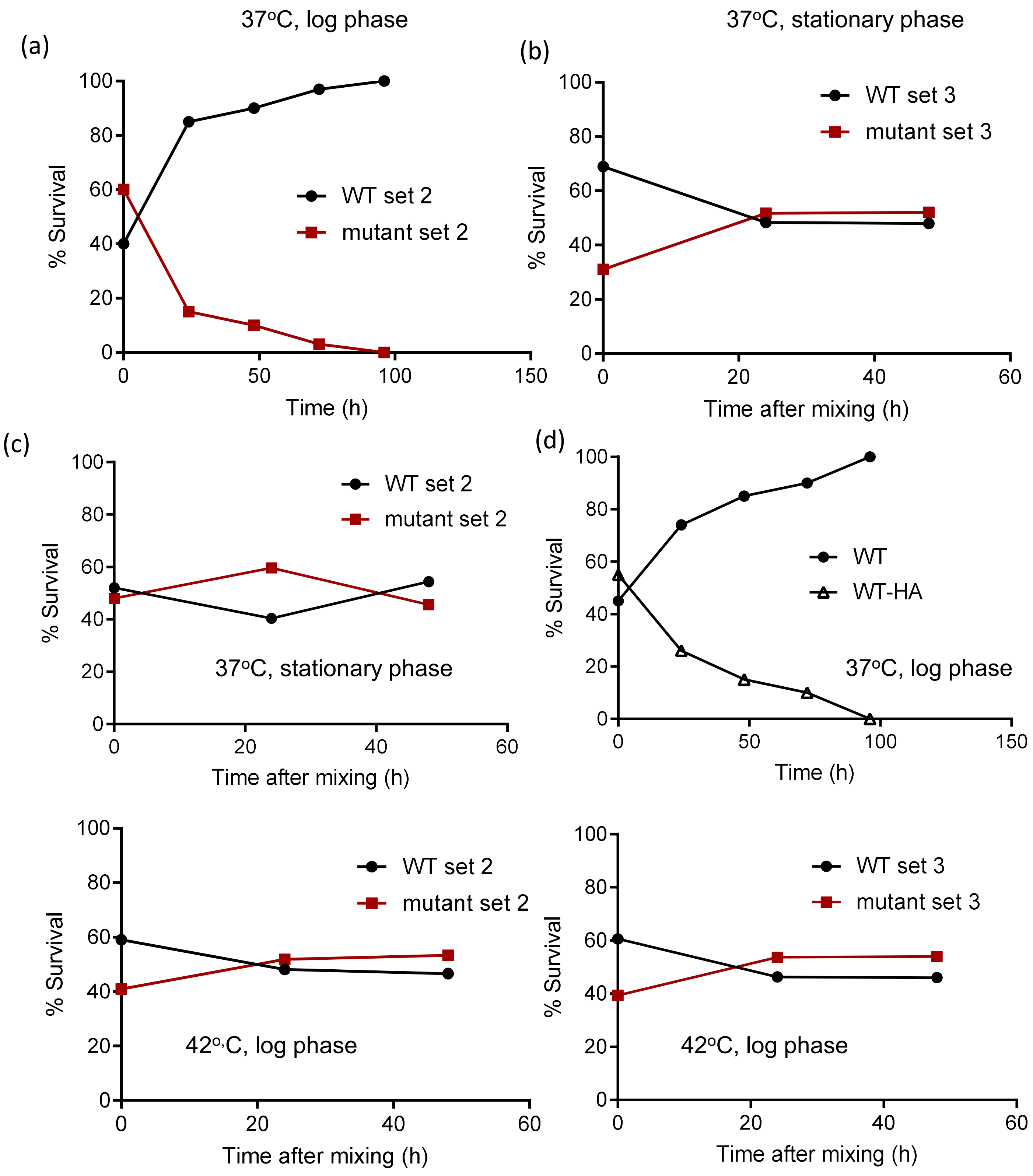

Figure S11. Replicate blocks for pair-wise competition experiments: Pair-wise growth competition experiments, strains and conditions as indicated, see Fig.4b-e.

Figure S12

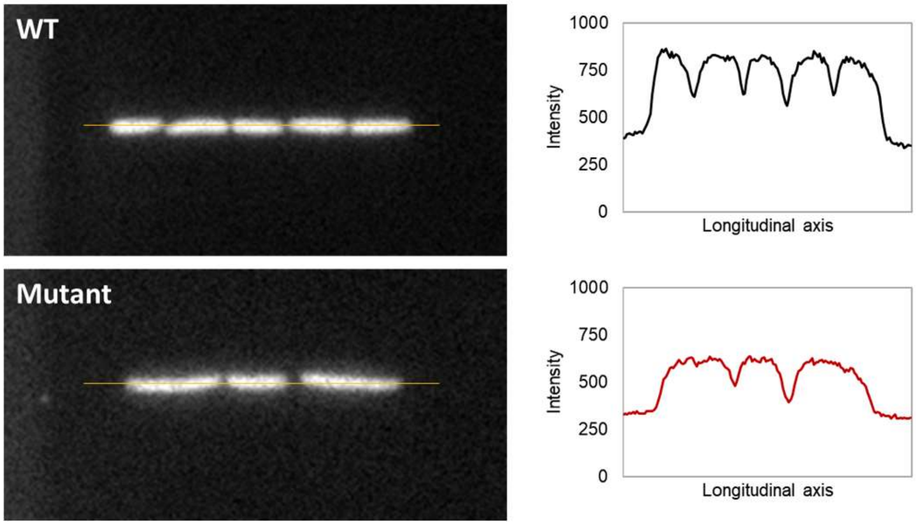

Figure S12: WT and Mutant cells within a representative single growth channel, with the fluorescence intensity plotted from the region of interest drawn across the cells.
